# Proximity induced band gap opening in topological-magnetic heterostructure (Ni_80_Fe_20_/p-TlBiSe_2_/p-Si) under ambient condition

**DOI:** 10.1038/s41598-023-49004-5

**Published:** 2023-12-15

**Authors:** Roshani Singh, Gyanendra Kumar Maurya, Vidushi Gautam, Rachana Kumar, Mahesh Kumar, K. G. Suresh, Brahmaranjan Panigrahi, Chandrasekhar Murapaka, Arbinda Haldar, Pramod Kumar

**Affiliations:** 1 Spintronics and Magnetic Materials Laboratory, Department of Applied Sciences, Indian Institute of Information Technology Allahabad, Prayagraj, 211015 India; 2 CSIR - Indian Institute of Toxicology Research, Lucknow, 226001 India; 3 CSIR-National Physical Laboratory, New Delhi, India; 4 Department of Physics, Indian Institute of Technology Bombay, Mumbai, 400076 India; 5 Department of Physics, Indian Institute of Technology Hyderabad, Kandi, 502284 Telangana India; 6 Department of Materials Science and Metallurgical Engineering, Indian Institute of Technology Hyderabad, Kandi, Telangana 502284 India

**Keywords:** Nanoscience and technology, Optics and photonics, Physics, Materials science, Materials for devices, Materials for optics, Nanoscale materials, Soft materials

## Abstract

The broken time reversal symmetry states may result in the opening of a band gap in TlBiSe_2_ leading to several interesting phenomena which are potentially relevant for spintronic applications. In this work, the quantum interference and magnetic proximity effects have been studied in Ni_80_Fe_20_/p-TlBiSe_2_/p-Si (Magnetic/TI) heterostructure using physical vapor deposition technique. Raman analysis shows the symmetry breaking with the appearance of A^2^_1u_ mode. The electrical characteristics are investigated under dark and illumination conditions in the absence as well as in the presence of a magnetic field. The outcomes of the examined device reveal excellent photo response in both forward and reverse bias regions. Interestingly, under a magnetic field, the device shows a reduction in electrical conductivity at ambient conditions due to the crossover of weak localization and separation of weak antilocalization, which are experimentally confirmed by magnetoresistance measurement. Further, the photo response has also been assessed by the transient absorption spectroscopy through analysis of charge transfer and carrier relaxation mechanisms. Our results can be beneficial for quantum computation and further study of topological insulator/ferromagnet heterostructure and topological material based spintronic devices due to high spin orbit coupling along with dissipationless conduction channels at the surface states.

## Introduction

Three-dimensional (3D) topological insulators (TI) are gapped bulk insulators having two-dimensional (2D) gapless Dirac cones on the surface. These 2D surface states (SS) are conducting due to topological invariants which exhibit time reversal symmetry (TRS)Z^1–3^. Some peculiar phenomena like the existence of Majorana fermions, quantum anomalous Hall effect (QAHE), and the topological magnetoelectric effect are featured by the surface states of topological materials^4,5^. Under time-reversal operation, the electron wave vector k and the spin will alter their sign. The 2D surface states of a TI material remain unaffected because the opposing spin channels are locked to their corresponding momenta, however, such symmetry can be destroyed in the presence of a magnetic field (PMF) or magnetic impurities^6,7^. The TRS breaking induced SS of TI materials leads to a variety of novel quantum phenomena like topological magneto electric effect and QAHE. These unique properties of TI materials open a new dimension in condensed matter physics and in developing low-power spintronic devices^8,9^.

It is possible to break TRS via magnetic ordering, which can open the gap owing to magnetic exchange coupling^10^. There are two well-known methods to achieve this: (1) by doping of magnetic element and (2) by fabricating heterostructure with magnetic material (magnetic proximity structure)^11,12^. However, the former method results in the generation of contaminated phases, heterogeneity and small exchange gap which lead to more scattering in the specimen. Consequently, experimental realization of QAHE is only possible at extremely low temperature^13,14^. Therefore, it is quite interesting to fabricate a 3D TI and a ferromagnet (FM) heterostructure in which there are no antisite defects in bulk states as reported by Jieyi Liu et al.^15^. In such a TI/FM heterostructure, the TI is magnetized by the FM through the proximity effect, which has already been proposed by Huang et al.^16^. Magnetic proximity effect has several benefits over magnetic doping, i.e., the realization of the half-integer QAHE, switching the gap between surface states, and maintaining the intrinsic crystalline phase of topological material etc.^17,18^. Moreover, as suggested by Huang et al.^19^ and Li et al.^20^, the magnetic proximity effect provides a higher temperature for magnetization survival that could be useful for spintronic applications. Hence, the magnetic proximity effect is a better method to create the gap between the surface states of TI materials. The gap between the surface states can be observed through angle-resolved photoemission spectroscopy (ARPES). Although, ARPES can measure the electronic structure of materials down to a few nm depth of the surface, the magnetic proximity effect occurs at the interface of TI/FM heterostructure making this technique unfit. Therefore, researchers have focused on different methods to observe gap opening in surface states, such as magnetoresistance (MR) measurement in which analysis of conductivity vs. magnetic field can give information about gap opening^21,22^. MR describes how resistance changes with an external magnetic field i.e., $defined \,as=({R}_{B}-{R}_{0})/{R}_{0}]\times 100$^23^.

Due to interaction in distinct scattering loops in weakly perturbed electronic systems, different transport characteristics takes place, which can be easily realized experimentally in thin films^24^. The result shows that the surface state of topological material always exhibits weak anti-localization (WAL) behavior due to destructive interference of time reversed scattering loops caused by spin–orbit coupling^25^. However, the electrons exhibiting constructive quantum interference between time reversed scattering loops give a negative quantum correction to conductance, known as the weak localization (WL) effect^26,27^. In PMF, the positive MR is an interesting phenomenon indicating gap opening at the Dirac point of the topological surface states and dominance of the WL effect^27^.

In this work, we have successfully fabricated good quality p-TlBiSe_2_/Si and p-TlBiSe_2_/Ni_80_Fe_20_/Si heterostructures. Raman and transient absorption spectroscopy (TAS) studies have been carried out in order to investigate phonon vibrations and charge carrier dynamics, respectively. Under PMF, the ground state splitting due to symmetry breaking is observed in TASs studies. Electrical analysis is done under dark and illumination conditions in both AMF (absence of magnetic field) and PMF. The impact of magnetic field on the TI material was also investigated in detail to explore various quantum interference phenomena (WAL and WL) using magnetoresistance measurement. Furthermore, we have investigated magnetic proximity effect in p-TlBiSe_2_/Ni_80_Fe_20_/p-Si heterostructure via MOKE measurement. All these measurements give an indication of the gap opening in PMF.

## Results and discussion

### XRD analysis

In order to investigate the structural analysis and physical properties of p-TlBiSe_2_ film and p-TlBiSe_2_/Ni_80_Fe_20_ film, both deposited on Si substrate, the x-ray diffraction (XRD) analysis was carried out, which confirms the polycrystalline nature of grown films. The XRD analysis of p-TlBiSe_2_/p-Si film shows 3 high intensity peaks having crystallographic phase (001), (002), (112). While TlBiSe_2_/Ni_80_Fe_20_/p-Si film shows one additional peak at (111) (Figure S1). The additional peak is Ni_80_Fe_20_ film peak, confirmed by previously reported results^28^ From Figure S1 it is clear that, in TlBiSe_2_/Ni_80_Fe_20_/p-Si film spectra, p-TlBiSe_2_/p-Si film peaks are shifted towards smaller angle. This shifting occurs due to strain effect at the interface of TlBiSe_2_ and Ni_80_Fe_20_.

### Raman spectroscopy study

**Figure 1:**
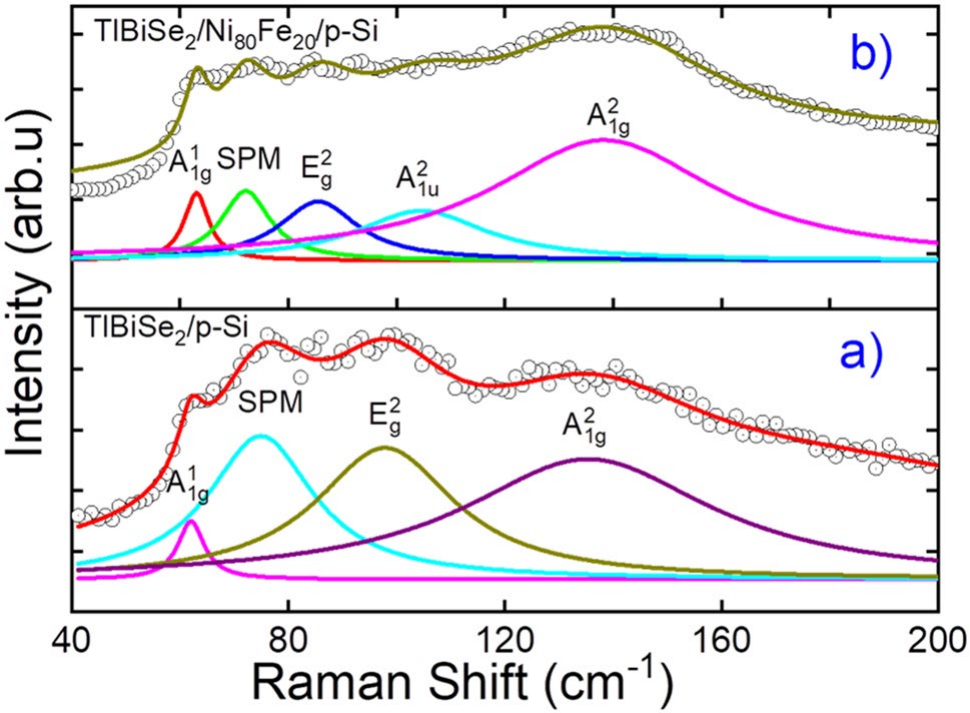
The Raman spectra. (**a**) p-TlBiSe_2_/p-Si heterojunction (**b**) p-TlBiSe_2_/Ni_80_Fe_20_/p-Si heterojunction.

Raman spectroscopy study of p-TlBiSe_2_/p-Si and p-TlBiSe_2_/Ni_80_Fe_20_/p-Si heterostructures was carried out to investigate the electron–phonon interaction and crystalline phases as shown in Fig. 1. TlBiSe_2_ exhibits rhombohedral crystal structure [as shown in Figure S1] of the space group R3m, and it has 15 phonon branches in which 12 are optical modes, and 3 are acoustic modes^29^, however, Ni_80_Fe_20_ exhibits the face-centred cubic (FCC) structure. In TlBiSe_2_/p-Si heterostructure, Raman active modes (A^1^_1g,_ E^2^_g,_ A^2^_1g_) along with surface phonon mode (SPM) at 74.91 cm^-1^ were observed, in good agreement with previously reported results^29^. The A^1^_1g_ (~ 61.99 cm^-1^), A^2^_1g_ (~ 135.3 cm^-1^) modes represent the “out of plane vibration” while E^2^_g_ ((~ 97.85 cm^-1^) mode represents the “in-plane vibration” with respect to the plane of covalent bonded quintuple layers of TlBiSe_2_. The emergence of SPM is due to the high surface-to-bulk ratio^30^. In p-TlBiSe_2_/Ni_80_Fe_20_/p-Si heterojunction, along with Raman active mode and SPM, an additional mode A^2^_1u_ is present, which is otherwise a forbidden mode. The emergence of this mode indicates the symmetry breaking of TlBiSe_2,_ which is due to the conjugation of Ni_80_Fe_20_ with TlBiSe_2_^31^. In p-TlBiSe_2_/Ni_80_Fe_20_/ p-Si, Ni_80_Fe_20_ magnetizes TI material, due to which spin momentum locking gets disturbed, resulting in the breaking of symmetry^32^.

From Table 1, it is clear that in p-TlBiSe_2_/Ni_80_Fe_20_/p-Si, the out-of-plane vibration (A^1^_1g_ ~ 63.09 cm-^1^, A^2^_1g_ ~ 138.32 cm^-1^) intensity has increased while “in-plane vibration intensity” (E^2^_g_ ~ 85.52) has decreased, indicating that, in the former, the “out plane vibrations” became less restrained or more active and the “in-plane vibrations” became more restrained due to symmetry breaking and weaker interaction between the layers. Therefore, the intensity ratio I(A^2^_1g_)/I(E^2^_g_) is enhanced in p-TlBiSe_2_/Ni_80_Fe_20_/p-Si heterostructure in comparison to that of p-TlBiSe_2_/p-Si.

**Table 1:** All the observed peaks are Lorentz fitted, and the corresponding data is listed.

Vibrational modes	p-TlBiSe_2_/p-Si			p-TlBiSe_2_/Ni_80_Fe_20_/ p-Si		
	Peak position (cm^-1^)	FWHM (cm^-1^)	I(A^2^_1g_)/I(E^2^_g_)	Peak position (cm^-1^)	FWHM (cm^-1^)	I(A^2^_1g_)/I(E^2^_g_)
A^1^_1g_	61.99 ± 0.32	5.92 ± 1.58	1.38	63.09 ± 0.44	5.45 ± 1.67	1.62
E^2^_g_	97.85 ± 0.84	31.10 ± 4.42		85.52 ± 1.68	17.63 ± 10.27	
A^2^_1g_	135.3 ± 1.47	55.95 ± 17.2		138.32 ± 1.81	50.79 ± 6.05	
SPM	74.91 ± 0.54	23.70 ± 3.69		72.14 ± 0.84	10.93 ± 4.69	
A^2^_1u_	–	–		104.51 ± 3.59	29.16 ± 16.29	

### Magnetic field induced transient absorption spectrpscopy (TAS) study

**Figure 2:**
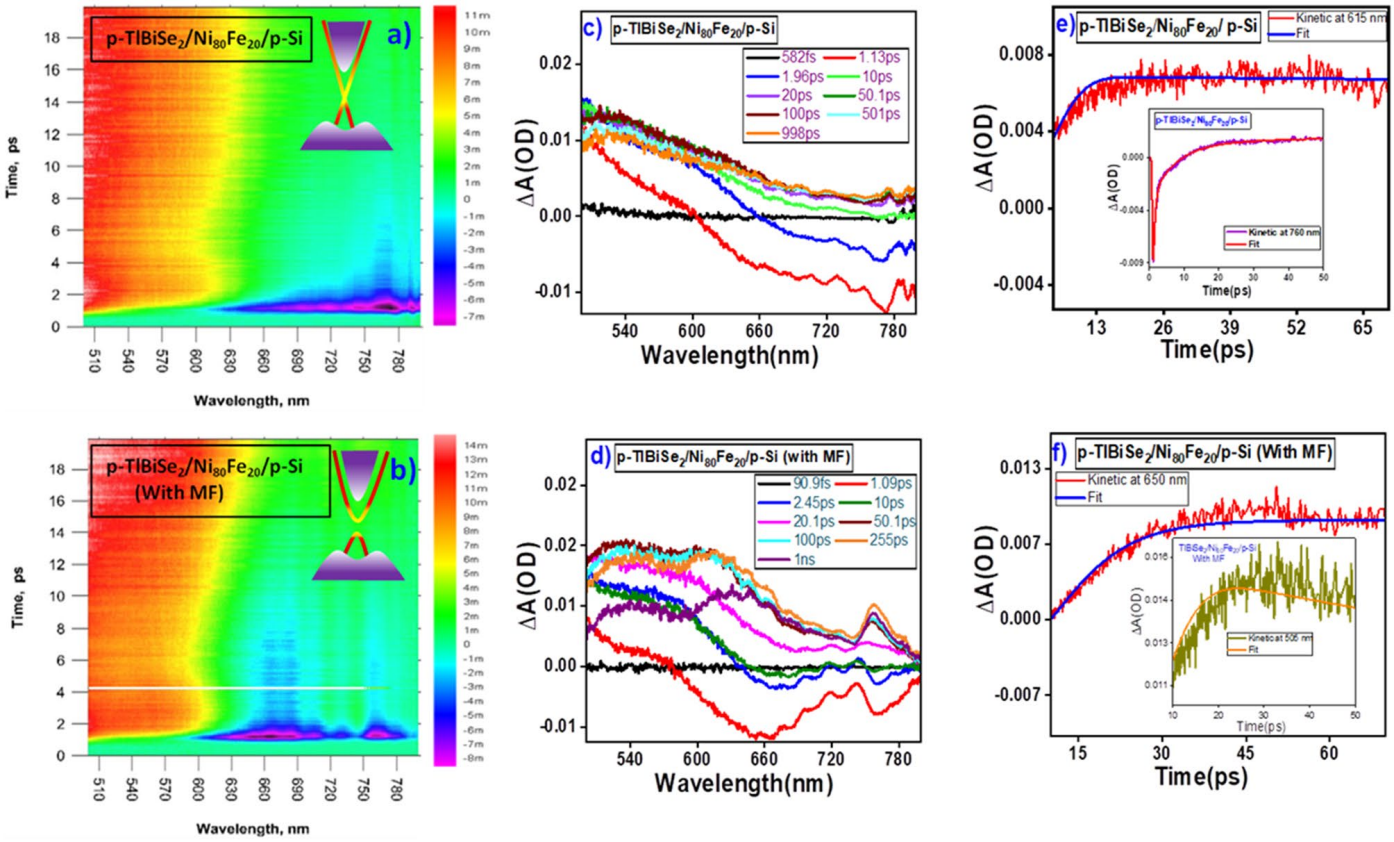
Ultrafast transient absorption spectra of p-TlBiSe_2_/Ni_80_Fe_20_/p-Si film from 400 to 800 nm wavelength in AMF and PMF. (**a**, **b**) show the ultrafast surface of examined heterostructure while corresponding insets represent TlBiSe_2_ band structure in AMF and PMF, respectively. (**c**, **d**) show TA spectra with varying probe delay in AMF and PMF, respectively. (**e**, **f**) show kinetic profiles in AMF and PMF, respectively. ( The representation of the heterostructure for this characterization is shown as Fig. 8 in experimental section).

The charge carrier dynamics of p-TlBiSe_2_/Ni_80_Fe_20_/p-Si heterostructure were recorded using ultrafast transient absorption spectroscopy in both AMF and PMF (~ 800 Oe). The result gives a complete study of charge carrier dynamics and phonon dynamics. The samples were excited using 410 nm pump wavelength with a power of 0.2 mW. Figure 2a and b exhibit ultrafast surfaces in the visible range for both AMF and PMF, respectively. From Fig. 2b, it is clear that in PMF, ground state energy level splitting takes place due to the Zeeman effect which is caused by the interaction between electrons magnetic moment and external magnetic field. Consequently, the gap at the Dirac point of TlBiSe_2_ opens (inset of Fig. 2b), leading to TRS breaking of TlBiSe_2_^33^ and the same is confirmed by electrical analysis also^34^.

Figure 2c and d show the ultrafast spectra in AMF and PMF, respectively. It is clear that AMF spectra shows broad ground state bleaching (GSB) from 600 to 800 nm having maximum bleaching at 770 nm for a very long time (3.6 ns) with some anti-Stoke shifted spectrum representing stimulated emission. The appearance of GSB is due to the movement of charge carriers from the valence band to conduction band^35^. The GSB is transformed to photo-induced absorption spectra in the 400 to 600 nm wavelength range exhibiting maximum absorption at 505 nm with a lifetime of 1.99 ns. The positive transient absorption spectra also appeared after the 1.96 ps delay of the probe having maxima at 775 nm wavelength.

From Fig. 2d, it is clear that, in PMF, GSB appears from 575 to 800 nm, having a maximum at 650 nm for a very short time (6.2 ps), and the stimulated emission spectra will also appear having a maximum at 760 nm. This GSB is transformed to photo-induced absorption spectra in the range of 400 nm to 575 nm with maximum absorption at 535 nm having small life time (ps order). Transient spectra again appear at 757 nm after 10 ps of probe delay with a lifetime of a few picoseconds.

To determine the lifetime of each spectrum, the kinetic profile for each signal is simulated using the phonon fitting model in the surface explorer software. With a sum of convoluted exponentials, the below function^36^ enables fitting a kinetic trace at the chosen wavelength.

1 $$S\left(t\right)= {{e}^{-}}^{({\frac{t-to}{tp})}^{2}}*\sum_{i}{A}_{i}{{e}^{-}}^{\frac{t-t0}{ti}}$$

where A_i_ is amplitude, t_i_ is decay time, t_0_ is zero time, and t_p_ is instrument response time. Here, we have fitted the kinetic spectrum using the triexponential decay function for various wavelengths and probe delays as shown in Fig. 2e and f. The corresponding decay times are listed in Table 2.

**Table 2:** List of derived parameters for p-TlBiSe_2_/Ni_80_Fe_20_/p-Si heterostructure from the kinetic fittings of TA spectra.

TlBiSe_2_/Ni_80_Fe_20_ /p-Si (in AMF)					TlBiSe_2_/Ni_80_Fe_20_ /p-Si (in PMF)				
Wavelength(λ) nm	E(ev) = 1240/ λ	τ_1_	τ_2_	τ_3_	Wavelength (λ)nm	E(ev) = 1240/λ	τ_1_	τ_2_	τ_3_
505 (TA) (1.96 ps)	2.45	257 ps	1.99 ns		505 (TA) (2.45 ps)	2.45	1.4 ps	15.8 ps	
615 (GSB) (1.33 ps)	2.02	43 ps	6.8 ps	510 fs	650(GSB) (1.04 ps)	1.91	6.2 ps	708.3 fs	6.2 ps
770 (GSB)	1.63	2.63 ns	2.26 ns	3.6 ns	760 (TA)	1.63	1.4 ps	15.86 ps	

From Table 2, it is clear that in PMF, decay time becomes shorter, indicating fast decay of charge carriers. Therefore, charge carriers’ decay in a very short time to lower energy states through phonon-mediated inter- and intra-band scatterings. In AMF, the charge carriers are stabilized due to inter transitions in higher energy states before relaxing back to lower energy states^37^.

### Magnetic field induced electrical analysis under dark condition

**Figure 3:**
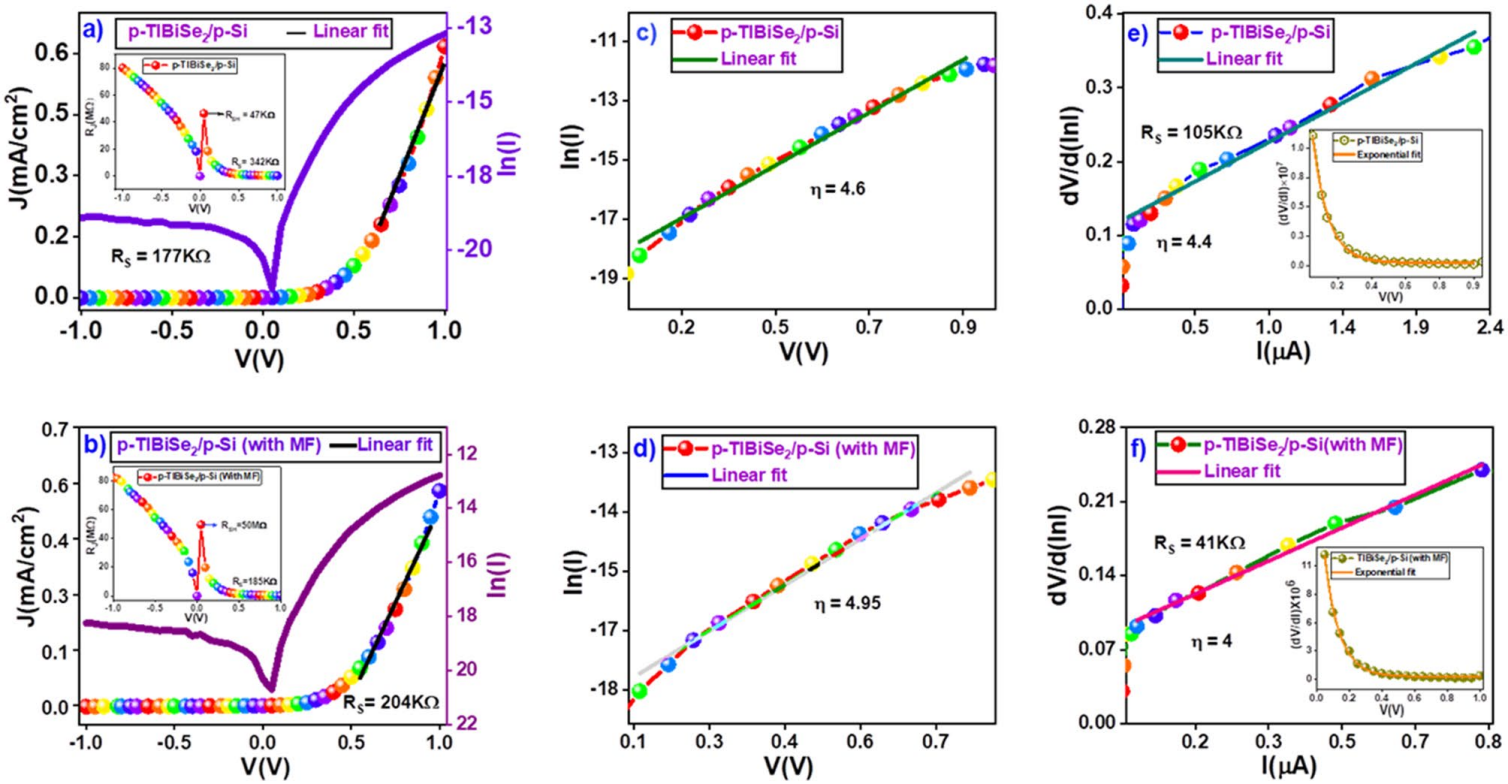
Dark characteristics of p-TlBiSe_2_/p-Si heterostructure at room temperature in AMF and PMF. (**a**, **b**) show the photocurrent density vs. voltage (J–V) plot and magnified semi-log characteristics plot, while the inset shows the resistance voltage (R–V) plot in AMF and PMF, respectively. (**c**, **d**) show the semi-log (I) *vs* V characteristics in AMF and PMF respectively. (**e**, **f**) shows the forward biased linearly fitted plot of $\frac{dV}{d\left(lnI\right)}$ *vs*. I while the corresponding inset shows the ($\frac{dV}{dI} )$ *vs* V characteristics, showing the exponential relation of the slope with applied voltage in AMF and PMF, respectively. (The representation of the heterostructure for this measurement is shown as Fig. 7 in experimental section).

Various essential junction parameters of heterojunction diode like rectification ratio, ideality factor, and built-in potential can be obtained from the I-V measurements^38^. The room temperature electrical measurements of p-TlBiSe_2_/p-Si heterojunction were carried out in both AMF and PMF. Figure 3a and b show I–V characteristics and reveled the nonlinear behavior of electric current with forward applied voltage in AMF and PMF, respectively. The examined devices exhibit an excellent rectification ratio (as listed in Table 3), which can be attributed to less leakage current (nA order) and high forward current (μA order). This demonstrates that the heterojunction device acts as a high-quality diode. The two primary factors impacting diode functionality are series resistance $({R}_{S}$) and shunt resistance (R_SH_). The greater the current flow through the heterojunction diode, the lower its series resistance. On the other hand, the higher shunt resistance reduces leakage current and optimizes the diode efficiency of the device. The value of ${R}_{S}$ and R_SH_ were calculated from the I–V outcomes of examined heterojunction device by using the relation ${R}_{S}= \frac{\partial V}{\partial I}$.

**Table 3:** Under the dark condition, the calculated diode parameters for p-TlBiSe_2_/p-Si, Ni_80_Fe_20_/p-TlBiSe_2_/p-Si, p-TlBiSe_2_/p-Si (magnetic film on the top surface) and p-TlBiSe_2_/p-Si (No magnetic film on p-TlBiSe_2_ surface) heterojunctions at room temperature: AMF (absence of magnetic field) and PMF (presence of magnetic field).

Device	Forward current I_F_ (μA)	Reverse current I_R_ (nA)	Rectification Ratio I_F_/I_R_	R_S_ (KΩ)	R_SH_ (MΩ)	η from slop of ln (I) vs V plot	η from Cheung’s method
p-TlBiSe_2_/p-Si (AMF)	3.03	0.013	245	177	50	4.6	4.4
p-TlBiSe_2_/p-Si (PMF)	2.1	0.012	239	185	47	4.95	4.8
Ni_80_Fe_20_/p-TlBiSe_2_/p-Si (AMF)	9.4	0.23	42	53	85	4	4.3
Ni_80_Fe_20_/p-TlBiSe_2_/p-Si (PMF)	8.2	0.53	15	58	80	5.13	5
p-TlBiSe_2_/p-Si (with Ni_80_Fe_20_ film)	3.03	0.013	245	177	50	4.6	4.4
p-TlBiSe2/p-Si	5	0.002	260	185	40	4	3.7

Inset of Fig. 3a and b show resistance *vs.* voltage plot, the value of ${R}_{S}$ and R_SH_ were tabulated in Table 3. The diode current of p-TlBiSe_2_/p-Si heterojunction can be obtained by the conventional diode equation^39^.

2 $$I={I}_{O }[\mathrm{exp}\left(\frac{qV}{\eta {k}_{B}T}\right)-1]$$

where, q is the electronic charge, V is the voltage applied, k_B_ is the Boltzmann constant, T refers to the temperature, I_0_ indicates the reverse saturation current and $\eta$ is the diode ideality factor, which gives the information that how much I-V experimental data is close to an ideal diode. For sufficient bias voltage $V\gg \frac{{k}_{B}T}{q}$, then value of the ideality factor can be obtained from Eq. (2) as

3 $$\eta =\left(\frac{q}{{k}_{B}T}\right)\left[\frac{dV}{d\left(lnI\right)}\right]$$

The value of ideality factor was calculated with the help of Eq. (3) by inserting the slope value of lnI vs. V plot as displayed in Fig. 3c and d. The calculated ideality factor of examined device p-TlBiSe_2_/p-Si was found to be greater than one indicating deviation from ideal diode behavior. The rise in ideality factor is due to interface layers, interface states, and series resistance^40^. Thus, the impact of series resistance cannot be ignored for the examined device. In such a scenario, for the determination of diode parameters, many models have been established for examining the series resistance impact, among which Cheung's approach is one of the best methods. The Cheung's functions could be given by the following relation^40^.

4 $$\frac{dV}{d\left(lnI\right)}=\frac{\eta {k}_{B}T}{q}+ I{R}_{S}$$

From above expression it is clear that, $\eta$ and R_S_ of diode are determined from the slope and the intercept of $\frac{dV}{d\left(lnI\right)}$ *vs* I plot respectively. Figure 3e and f show dV/dln(I) *vs* current plot of p-TlBiSe_2_/p-Si heterojunction. The calculated values of ${R}_{S}$ and $\eta$ are given in Table 3. These values of the ${R}_{S}$ and $\eta$ derived from the Cheung’s method are nearly equal to those obtained by fitting the diode using Eq. (2) of the p–n junction diode. The obtained value of the ideality factor is much higher than its ideal value (1) due to the rise in diffusion current with an increase in applied voltage or due to electron and hole recombination in the depletion zone^41^.

Again, using the same expression, the diode parameters were calculated for Ni_80_Fe_20_/p-TlBiSe_2_/p-Si heterojunction in AMF and PMF (Figure S5). The obtained results are tabulated in Table 3. Along with this, the effects of magnetic thin film on electrical properties of p-TlBiSe_2_/p-Si heterojunction (magnetic film is grown on top surface of TI film) were also investigated at ambient condition. The result reveals that magnetic film has a significant effect on the charge transport characteristics of the device. The outcomes of the device in both scenarios have been given in Table 3.

The obtained results (as given in Table 3) reveal that the magnetic field significantly affects the performance of heterojunction devices. Even a small magnetic field made changes in the electrical outcome of examined device. Also, Arakushan et al., experimentally verified that, as the magnetic field increases, charge carrier diffusion length reduces, resulting in a reduction in forward current^42^. A detailed explanation is given in the next section.

From Table 3, it can be also seen clearly that the coating of the magnetic film causes a reduction in current across the junction. Because of the magnetic proximity effect, the ferromagnetic material (Ni_80_Fe_20_) magnetizes the TI material. The non-zero magnetization in the topological magnetic interface leads to the generation of the spin–orbit coupling (SOC) induced field owing to spin aggregation at the interface. The spin Hall effect (SHE) and Rashba-Edelstein effect (REE) are the two mechanisms responsible for this phenomenon. SHE explains the mechanism by which a TI layer charge current develops into a spin current. This spin current is generated due to the asymmetric spin deflection caused by SOC^43^. On the other hand, REE often develops at the interface due to spatial inversion symmetry breaking that leads to the development of an internal electrical field at the interface of Ni_80_Fe_20_/p-TlBiSe_2_ having a direction perpendicular to the film plane^44^. Whenever, an in-plane charge current propagates across the TI/FM heterojunction, the conduction electrons close to the interface travel in the electrical field and are subjected to an effective magnetic field that is perpendicular to the direction of current. Such interfacial induced effective magnetic field is known as the Rashba field.

**Figure 4:**
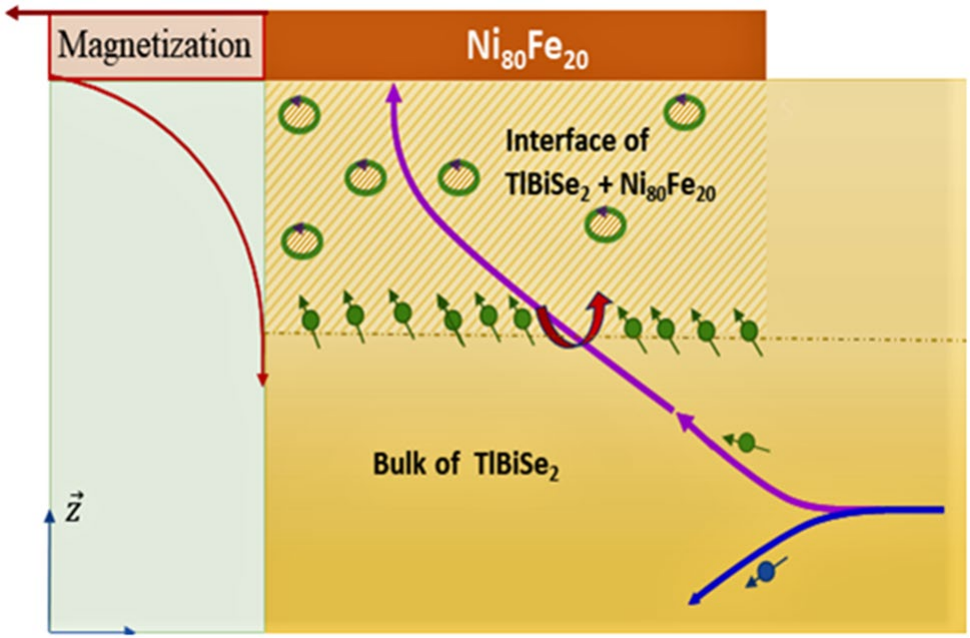
Schematic showing the generation of spin–orbit coupling and spin–orbit torque (SOT) in Ni_80_Fe_20_/p-TlBiSe_2_.

When a spin current flows across TI/FM heterojunction, the spin experiences a spin–orbit torque, (at the interface) which comprises mainly of two components i.e., longitudinal torque $({\tau }_{DL}$) and transverse torque (${\tau }_{FL}$). Both these are realized due to SHE and REE simultaneously. As the spins approach the interface, they experience torque resulting in the localization and randomization of spins, causing a reduction in the current (Fig. 4)^45^.

### Magnetic field induced charge transport study under light effect

The photodetection capabilities of p-TlBiSe_2_/p-Si heterojunction was also investigated under the illumination. The imposed laser light wavelength was varied from 500 to 1300 nm by TLS −300 XU Xenon light source. The optical power was kept at 2.96 μW throughout the investigation. Figure S6a and S6b show the I–V characteristic of examined heterojunction under light effect in AMF and PMF, respectively. The results demonstrate excellent photoelectric and photovoltaic effects in both forward and reverse bias regions. The photodetection efficiency of device was assessed through performance parameters like, photo to dark current ratio $PDCR= {I}_{ph}/{I}_{dark}$, photoresponsivity $R={I}_{ph}/{P}_{i}$, detectivity $D= R{A}^{1/2}/\sqrt{2q{I}_{dark}}$ , sensitivity $S=R(d/{V}_{d})$ and photoconductive gain $G= Rh\nu /q\eta$ ^46^. Where ${I}_{ph}=$ (${I}_{light}-{I}_{Dark}$) is photo current, I_dark_ is dark current, P_i_ represents the power density of laser light, A represents the effective area of the device for absorbing incoming light (0.0049 cm^2^) ,$\nu$ represents the frequency of the incoming laser light, h is the Planck’s constant , $\eta$ represents the external quantum efficiency, V_d_ is the bias voltage, and d is the thickness of the diode (~ 150 nm).

From Figure S6a and S6b, it is clear that the illumination current is significantly higher than the dark current, due to the generation of electron–hole pairs. As the incident light energy increases, the device current also increases up to 1.38 eV, corresponding to 900 nm, and a further increase in the incident light energy, photo response decreases due to the increase in the number of electron–hole pairs. The excess electron–hole pairs cause an enhanced scattering and therefore, local heat generation takes place that results in a decrease in the photo response^47^. Figure S6c and S6d show an E-V-R contour plot that revealed efficient absorption of incident photons. The maximum responsivity is obtained, corresponding to 1.38 eV. As TlBiSe_2_ is a narrow band gap (0.33 eV) material, the enhanced photo response is contributed by light absorption of the TI material. Figure S6e and S6f. show the photoconductive gain and detectivity as a function of wavelength, giving rise to a maximum response at 900 nm, while the inset figure shows the sensitivity variation for varying wavelengths as a function of voltage, which also gives maximum response corresponding to 900 nm. All the calculated performance parameters for the examined device p-TlBiSe_2_/p-Si at + 1 V in AMF and PMF are tabulated in Table 4. Similarly, for Ni_80_Fe_20_/p-TlBiSe_2_ heterojunction, all the photo detection parameters were calculated using the above expressions. Here also, a significant reduction in photocurrent was observed in PMF. The photocurrent characteristic of Ni_80_Fe_20_/p-TlBiSe_2_heterojunction with varying energy of incident light is shown in Figure S7. The examined heterostructures demonstrated maximum photo response corresponding 1.37 eV to and 2.08 eV and the calculated parameters are listed in Table 4. In PMF photo detection capability of the device is significantly reduced. The rational for this observation is discussed in the section below.

**Table 4:** List of photo detection parameters at maximum operating wavelength for p-TlBiSe_2_/p-Si and Ni_80_Fe_20_/p-TlBiSe_2_ heterojunction in AMF and PMF.

Device	Wavelength (nm)	Forward current (I_F_)(µA)	PDCR	R(A/W)	Gain	D(cm Hz1/2/W) × 10^11^	S (m/ΏW) × 10^–6^
p-TlBiSe_2_/p-Si (AMF)	900	3	7.74	7.94	10.93	5.65	1.92
p-TlBiSe_2_/p-Si (PMF)	900	2.27	3.91	3.75	5.78	2.90	0.55
Ni_80_Fe_20_/p-TlBiSe_2_ (AMF)	600	411	–	141	290	12.25	–
Ni_80_Fe_20_/p-TlBiSe_2_ (PMF)	600	122	–	100	280	9.12	–

### Magnetic induced effects responsible for current reduction

The 3D topological material contains an odd number of mass-less 2D Dirac cones. These Dirac cones have helical spin structures. When an electron moves around the Fermi surface, they follow time reversed scattering loops owing to the generation of π Berry phase. The generation of this Berry phase results in the absence of backscattering due to an additional phase factor^48^. Therefore, delocalization of surface charge carriers of TI material takes place which is experimentally confirmed by weak antilocalization effect. Such a peculiar phenomenon is realized due to the destructive interference of time reversed loops, which nullifies the backscattering probability of carriers^49^. On the other hand, under a magnetic field, time reversed scattering loops exhibit constructive interference, yielding to an enhancement in the backscattering probability of charge carriers causing an increase in resistivity^50^. The following section elucidates the fundamental quantum interface effects (WAL and WL) in TI materials that significantly affect conductivity. The charge transport quantum diffusion mechanism is a foundation to grasp quantum interference effects (WAL and WL) in TI materials.

The charge transport in solids depends on several factors like mean free path (*l*), phase coherence length (${l}_{\varnothing }$) and size of sample (L). If *l* >  > L, the specimen permits charge carriers tunneling without scattering termed as ballistic transport mechanism. Contrary to this, the diffusive transport mechanism exists when *l* <  < L, in this scenario, the charge carriers are scattered and dispersed across the specimen. The diffusive regime with ${l}_{\varnothing }>>l$ condition, called quantum diffusive regime, in which the charge carriers maintain their phase despite multiple scatterings. This phenomenon exists specially in the surface state of topological insulators where quantum interference in time reversed scattering loops occurs, introducing WAL and WL effects^51^. Such an effect significantly changes the conductivity of TI material that is given by $\upsigma \propto \left(\pm \frac{{\mathrm{e}}^{2}}{\mathrm{\pi h}}\mathrm{ ln}\frac{{\mathrm{l}}_{\mathrm{\varnothing }}}{\mathrm{l}}\right)$^51^, where $\frac{{e}^{2}}{h}$ is known as the quantum conductance and ( +) and (–) sign are for WAL and WL, ${l}_{\varnothing }$ is calculated from inelastic scattering due to electron–phonon or electron–electron scattering^52^.

The external magnetic field causes TRS breaking of TI material. Consequently, ${l}_{\varnothing }$ is reduced while the mean free path remains constant, resulting in the reduction of conductivity according to the above expression^51^. This is further experimentally confirmed by the MR measurement and MOKE measurement.

### Experimental realization of WL predominance in the presence of magnetic field

The magnetoresistance behavior of a TlBiSe_2_/Ni_80_Fe_20_/p-Si film is influenced by the properties of both materials (TlBiSe_2_ & Ni_80_Fe_20_) and their interfaces. When TlBiSe_2_ is deposited over Ni_80_Fe_20_, the ferromagnetic properties of Ni_80_Fe_20_ and the unique electronic states of TlBiSe_2_ can interact. The magnetization dynamics in Ni_80_Fe_20_ have the potential to influence the magnetic field experienced by the TlBiSe_2_ layer, thereby influencing the cyclotron motion of charge carriers within the topological surface states. The observed magnetoresistance behavior in TlBiSe_2_/Ni_80_Fe_20_/p-Si heterostructure can be attributed to the combined effect of quantized cyclotron orbits in TlBiSe_2_ and the magnetic properties of Ni_80_Fe_20_. The variations in the magnetic field can potentially induce changes in the cyclotron motion of charge carriers within TlBiSe_2_, thereby having an influence on the overall resistance of the heterojunction.

**Figure 5:**
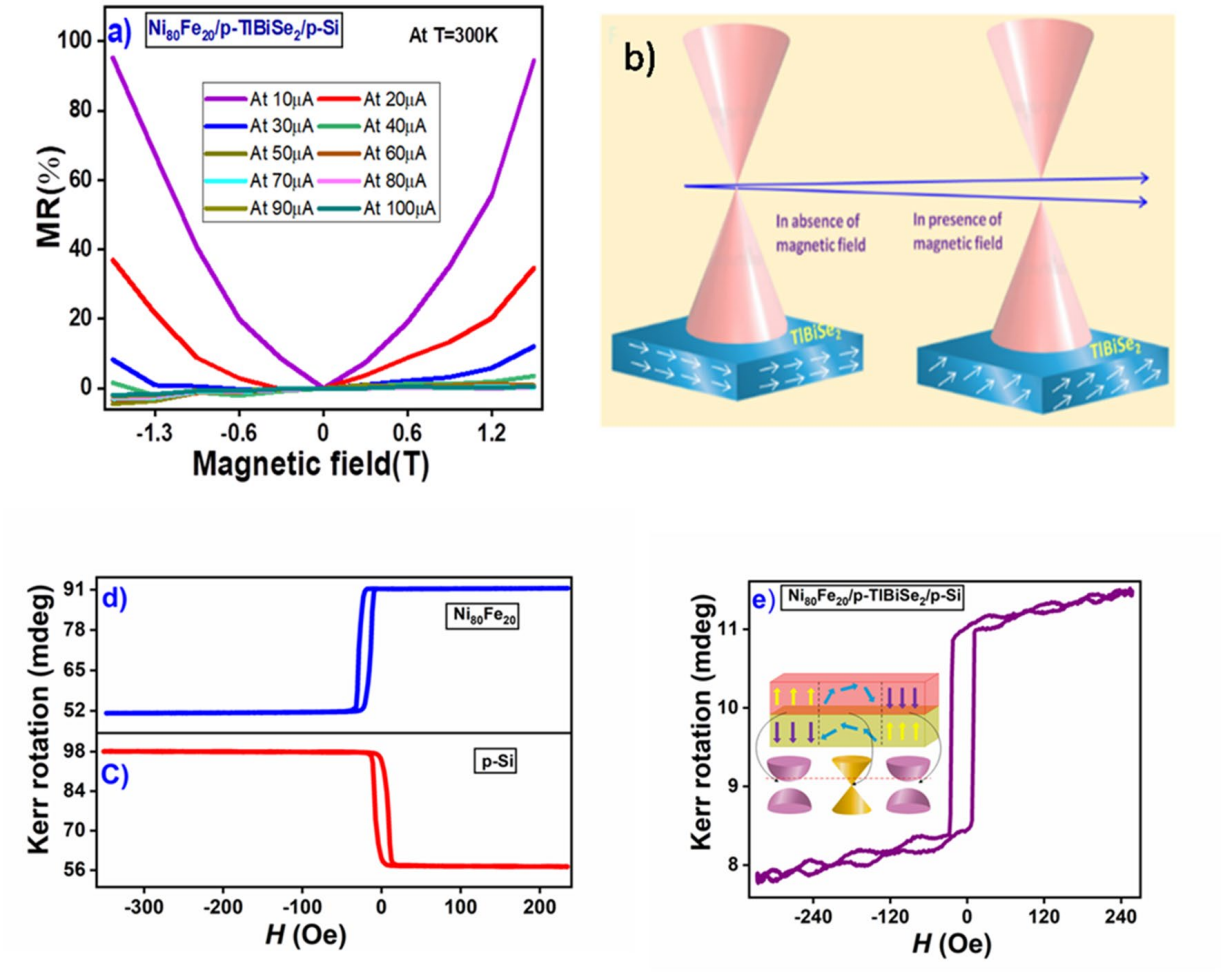
(**a**) MR vs. magnetic field plot for various currents. (**b**) Schematic of the band gap opening in TI due to TRS breaking. (**c**, **d**, **e**) shows the room temperature MOKE hysteresis loops of p-Si, Ni_80_Fe_20_ and Ni_80_Fe_20_/p-TlBiSe_2_/p-Si, respectively. While the inset of (**e**) shows the cross–sectional view of Ni_80_Fe_20_/p-TlBiSe_2_.

Futhermore in TI materials, WAL effect originates due to the strong SOC and spin-momentum locking in the surface states. While in PMF, domination of WL and separation of WAL occur simultaneously. The crossover of WL is a clear evidence of TRS breaking and the appearance of a topological gap in surface states. Figure 5a shows the percentage MR of Ni_80_Fe_20_/p-TlBiSe_2_/p-Si heterostructure measured at different constant currents. The examined heterostructure exhibits a positive MR, showing a maximum value of 95% at 10µA current, corresponding to 1.5 T. Interestingly, near zero magnetic fields, MR displays a pronounced sharp cusp pattern which is the hallmark of the WAL effect. Moreover, as the magnetic field increases, the cusp characteristic disappears, indicating the dominance of WL. However, this cusp forfeited its sharpness as current is increased and almost disappeared for higher than 40µA current. This is most probably due to the reduction in phase coherence length owing to the excess electron–phonon interaction^17^. As we increase the magnetic field, the MR also increases due to the transition from WAL to WL. This transition suppresses the topological protection of surface states and leads to the TRS breaking in the TI material, which in turn results in the gap opening at the Dirac point in the quantum diffusive regime^53^ as shown in Fig. 5b.

In order to verify the broken TRS in the TI material and the magnetic characteristics of the topological magnetic heterostructure, the magnetization reversal process was measured using the magneto-optical Kerr effect (Durham Magneto Optics: NanoMOKE3), where the magnetic field was varied in a range of ± 3500 Oe. From Fig. 5c and d it is clear that the MOKE signal of p-si and Ni_80_Fe_20_ are opposite in direction (sign) with different magnitude. Therefore, the net magnetization of p-Si/Ni_80_Fe_20_ is negative. On the other hand, MOKE signal of Ni_80_Fe_20_/p-TlBiSe_2_/p-si is positive (Fig. 5e). The results reveal that the TlBiSe_2_ layer contributed to the MOKE signal, due to proximity effect at the topological magnetic interface. It can be understood via exchange interaction, which results in the spin-polarized state on the TlBiSe_2_ side. This spin polarization state is in the opposite direction of the Ni_80_Fe_20_ magnetization which was also theoretically confirmed by Eremeev et al.^54^.

To break the TRS of TI material, the Ni_80_Fe_20_ must have an out-of-plane component of the magnetization, which is already present here due to the canting of the magnetization^55^. Hence, the gap between the SS state of TI material is induced due to proximity-induced magnetic ordering caused by the exchange coupling between the TlBiSe_2_ top surface and the bottom surface of Ni_80_Fe_20_.Also at applied fields below the starting field, there is a coupling between TlBiSe_2_ and the perpendicular component of Ni_80_Fe_20_ magnetization. Where the domain walls in Ni_80_Fe_20_ results in a gapless region in the SS as shown schematically in the inset of Fig. 5e. The results corroborate well with tha MR study.

### Ni_80_Fe_20_/p-TlBiSe_2_/p-si heterojunction band diagram

**Figure 6:**
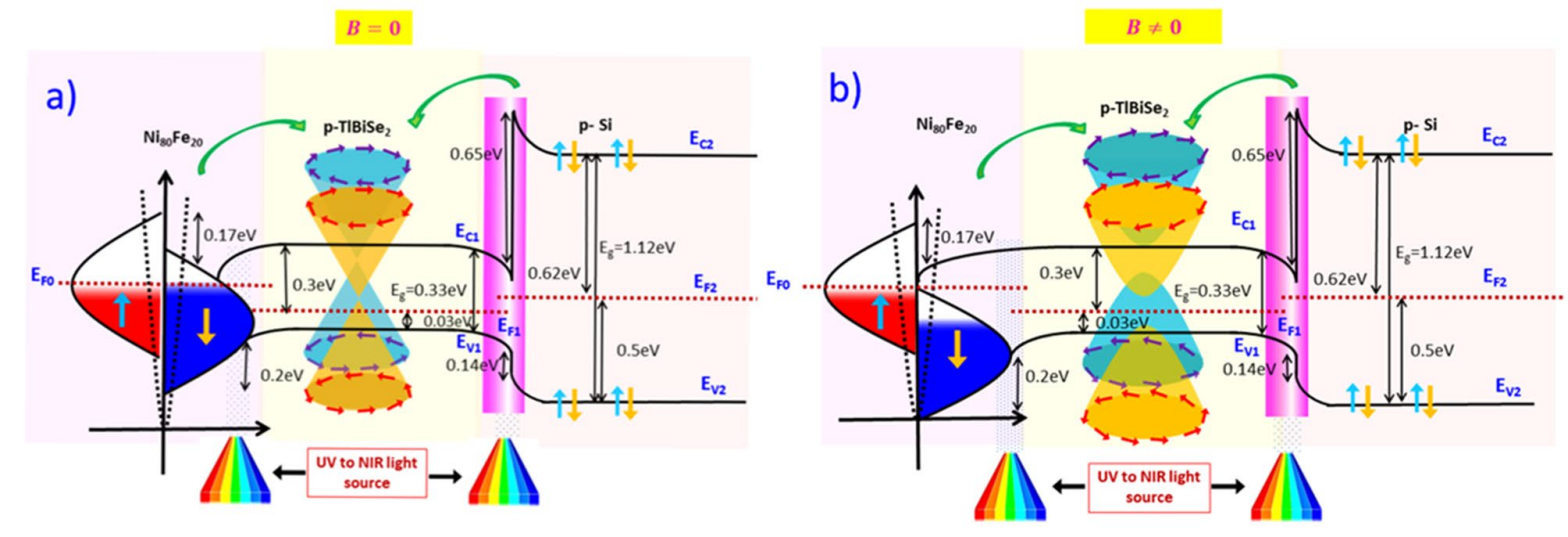
Schematic of the energy band diagram of the Ni_80_Fe_20_/p-TlBiSe_2_/p-Si heterostructure. (**a**) In AMF, under forward bias with light illumination resulting an increase in forward current. (**b**) In PMF, under forward bias with light illumination resulting decline in current due to TRS breaking in TI material.

An energy band diagram is useful to elucidate the charge transport mechanism and generation of photocurrent under light in the examined heterojunctions. Figure 6 depicts the band diagram of the Ni_80_Fe_20_/p-TlBiSe_2_/p-Si heterojunction at forward bias under light illumination in AMF and PMF. The abbreviations E_c_ and E_V_ are chosen to represent the appropriate conduction and valence bands, with suffixes 1 and 2 representing p-TlBiSe_2_ and p-Si, respectively. E_g_ represents the band gap of the material (E_g_ ~ 0 eV for Ni_80_Fe_20_, E_g_ ~ 0.33 eV for TlBiSe_2_, and E_g_ ~ 1.12 eV for Si). ΔE_C_, and ΔE_V_ are the conduction and the valence band offsets, which will act like a barrier in the flow of current. For Ni_80_Fe_20_/TlBiSe_2_ heterojunction ΔEc, ΔE_V_ are 0.17 eV and 0.20 eV, respectively, while for p-TlBiSe_2_/p-Si heterojunction the corresponding values are 0.65 eV 0.14 eV. The smaller band offset in Ni_80_Fe_20_/p-TlBiSe_2_ heterojunction is due to almost similar values of electron affinity of both the materials showing ohmic nature at the junction. In thermal equalibrium condition, the Fermi level of all three materials is aligned owing to diffusion of excess carriers from higher concentration region to lower concentration region. In the forword bias condition the shifting of the Fermi level occurs due to the applied bias. In Ni_80_Fe_20_/p-TlBiSe_2_ heterojunction, the Fermi level of Ni_80_Fe_20_ shifts towards the lower side while that of p-TlBiSe_2_ shifts towards the upper side. Similarly, in p-TlBiSe_2_/p-Si heterojunction, the Fermi level of p-Si has shifted towards the lower side while for p-TlBiSe_2_, it has shifted towards the upper side.

Under illumination and in AMF, the forward-biased Ni_80_Fe_20_/p-TlBiSe_2_/p-Si heterojunction is depicted in Fig. 6a. The incident light produces excess charge carriers, resulting in an increase in the current, which was also confirmed by electrical analysis. However, under magnetic field application, the spin-momentum locking in surface states of TI material gets affected, resulting in TRS breaking and gap opening at the Dirac point, as shown in Fig. 6b. Also, the magnetic field shifts the Fermi level of TI to close to the Dirac point (in actual topological materials, it is located at the valence band or conduction band). This shifting in the Fermi level alters the Berry phase of TI material which protects topological surface states from backscattering. It can be expressed by following equation^56^.

$${\varnothing }_{b}= \pi (1-\frac{\Delta }{{2E}_{F}})$$

where $\Delta$ is the gap between the Dirac cones. In AMF, $\Delta =0 and hence$ from the above equation, the Berry phase (${\phi }_{b})=\pi$^57^, which results in the absence of backscattering and delocalization of electrons. While in PMF, the Fermi level reaches very close to the Dirac point, the Berry phase is zero (${\phi }_{b})=0$ resulting in an enhanced backscattering probability and localization of electrons^58,59^.

## Conclusion

In conclusion, we have successfully grown magnetic-topological Ni_80_Fe_20_/p-TlBiSe_2_/p-Si heterostructure using the PVD technique. XRD analysis shows that the results are consistent with previously reported results. To investigate the phonon vibration, Raman analysis was carried out, which indicates TRS breaking through appearance of Raman forbidden mode. The electrical characteristics were investigated under dark and illumination conditions in AMF and PMF. The outcomes of the examined device reveal excellent photo response in both forward as well as reverse bias regions. Interestingly, under magnetic field, device shows a reduction in electrical conductivity at ambient condition. This decrease in conductivity is due to the transition from WAL to WL. The electrons which are in the surface states of the TI material, have π Berry phase leading to a destructive interface of time reversed scattering loops of the TI material, resulting in WAL. In PMF, due to exchange interaction at the interface, the Berry phase changes, resulting in a separation of the WAL. The crossover of WAL to WL was also experimentally confirmed by MR measurement, which was done by varying the field from −1.5 to 1.5 T. At zero magnetic field, WAL cusp is found, which disappears with increasing field, indicating the dominance of WL and hence increase in resistivity of the device.

Our results can be beneficial for quantum computation and further study of topological insulator/ferromagnet heterostructure and topological material based spintronic devices due to high spin orbit coupling along with dissipationless conduction channels at the surface states.

### Experimental section

Ni_80_Fe_20_ material were deposited on Si (100) substrate having a dimension of 1 × 1 cm^2^ via confocal DC magnetron sputtering set-up at room temperature where the target guns were oriented at an oblique angle to the substrate holder. Before the deposition process, the substrates were well cleaned with acetone and ethanol, followed by rinsing with di-ionized water. The substrates were mounted at the centre of the water-cooled sample holder, and the rotation of the substrate holder was performed at a 10 rpm speed to find the uniform thickness of the film. Prior to deposition, the Ni_80_Fe_20_ target (99.99% purity) was sputter-cleaned for 2 min to remove any surface contamination. The thickness of the Ni_80_Fe_20_ i.e., Permalloy layer was kept constant at 30 nm for all the samples. The base pressure of the chamber was maintained better than 5 × 10^−5^ Pa. In the sample growth process, the deposition pressure and sputtering power were maintained at 0.5 Pa and 80 W, respectively. In the obtained film of Ni_80_Fe_20_/p-Si, TlBiSe_2_ is deposited on the top surface of Ni_80_Fe_20_. The bulk form of 99.99% pure TlBiSe_2_, bought from Sigma Aldrich was used as the precursor to fabricate a thin film of TlBiSe_2/_Ni_80_Fe_20_/p-Si employing the thermal evaporation process (12A40D model manually operated) at room temperature under high vacuum (1.3 × 10^–4^ Pa). By using a diffusion pump, high vacuum within the chamber is created after that the Nickel boat (melting point of Ni = 1728 K) containing a solid precursor of TlBiSe_2_ passed an electric current of amount 60–70 A for 10 min. The fabricated film TlBiSe_2/_Ni_80_Fe_20_/p-Si is ready for the XRD, RAMAN, Transient absorption study, magnetoresistence measurement and MOKE measurement and the schematic 2 is shown below.

Now for the device characterization Ni_80_Fe_20_/p-TlBiSe_2_/p-Si heterostructure is fabricated. To obtain this heterostructure firstly p-TlBiSe_2_ is deposited on p-Si substrate via using thermal evaporator. Before the deposition process p-Si substrate was masked from all four sides using the aluminium foil and all the deposition parameter and process was kept same which was explained above. Now in obtained p-TlBiSe_2_/p-Si heterostructure Ni_80_Fe_20_ was deposited on top half surface of p-TlBiSe_2_ using confocal DC magnetron sputtering set-up at room temperature and again deposition process & parameters was kept same as earlier. As the Ni_80_Fe_20_ film is deposited on half surface of p-TlBiSe_2_ so hence before deposition remaining half surface of p-TlBiSe_2_/p-Si is masked by aluminium foil. To accomplish the Ni_80_Fe_20_/p-TlBiSe_2_/p-Si heterojunction, the aluminum foil utilized as a mask was detached after the deposition and the fabricated heterostructure is ready for electrical analysis.

**Figure 7:**
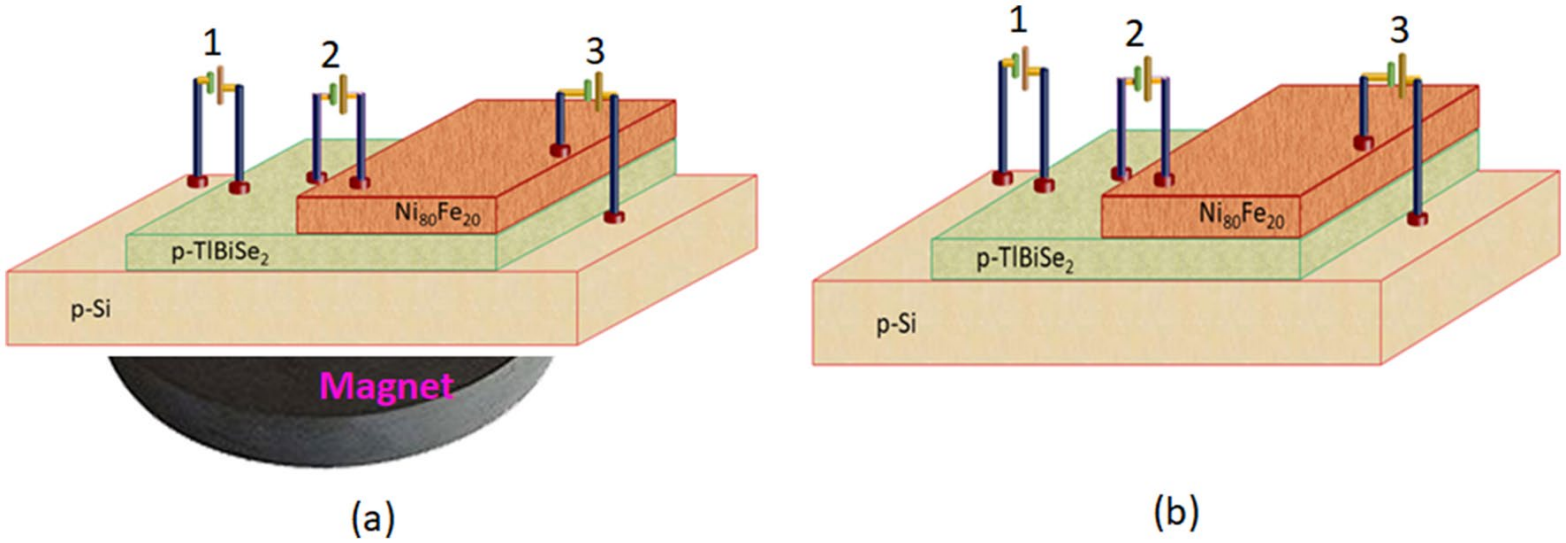
Schematic of Ni_80_Fe_20_/p-TlBiSe_2_/p-Si heterostructure in presence and absence of magnetic field respectively.

**Figure 8:**
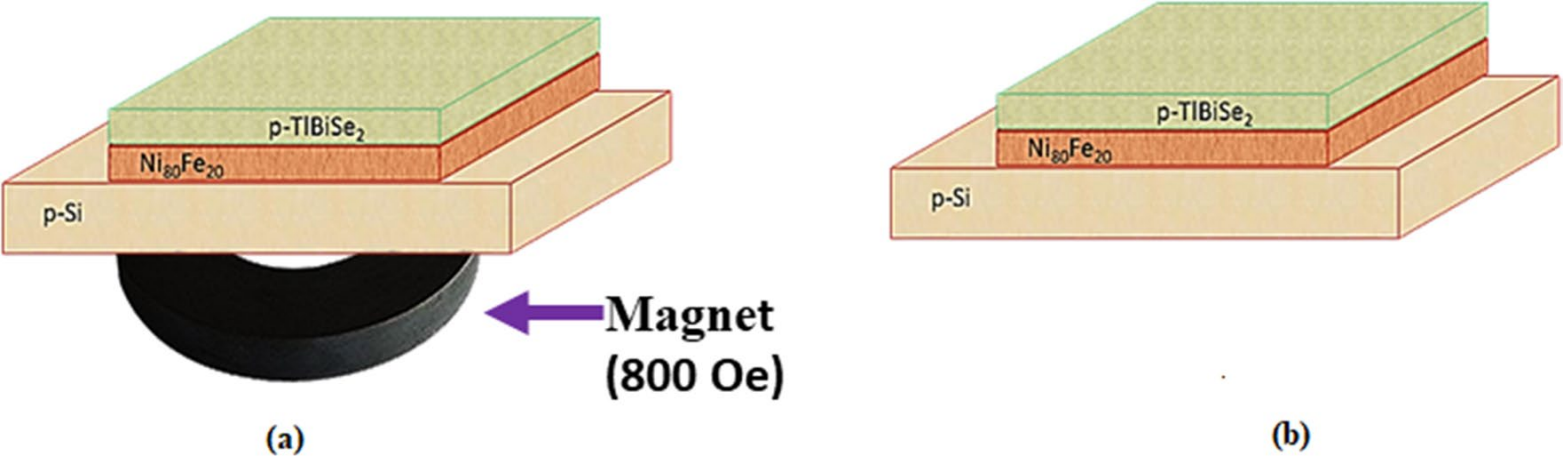
Schematic of TlBiSe_2/_Ni_80_Fe_20_/p-Si heterostructure in presence and absence of magnetic field respectively.

There are two types of schematics in the experimental section. One is for device fabrication purpose as shown in Schematic (as shown in Fig. 7) and another one is characterization purpose (XRD, RAMAN, Transient absorption study, magnetoresistence measurement and MOKE measurement) shown in Schematic (as shown in Fig. 8).

### Contact growth

After the fabrication of the Ni_80_Fe_20_/p-TlBiSe_2_/p-Si heterojunction, metallic contacts were grown by using same method as film deposition by thermal evaporator. To transfer the metallic contacts over the Ni_80_Fe_20_/p-TlBiSe_2_/p-Si film, a special hard mask with circular holes that were 300 µm in diameter and 700 µm apart were precisely positioned. The thermal evaporator method is used to evaporate pure Al wire (purity 99.99%) in a spiral tungsten (W) boat at a high vacuum (1.3 × 10^–4^ Pa) to create aluminum (Al) contacts. The thickness of the deposited contact was measured using an ellipsometer which was found to be 150 nm.

To determine the crystallite orientation and structural characteristics of the thin film XRD was performed (Rigaku Miniflex, Model No. BD68000014-01). The working conditions were: diffraction angle ranging from 10 to 70 with a step of 0.2; voltage = 40 kV; electric current = 15 A with copper (Cu) source (wavelength = 1.54). The I–V experiment was carried out using Keithley 4200 analyzer and the optoelectrical characteristic was investigated by stimulating the examined heterojunction with a TLS-300XU Xenon source of light at varying wavelength. The electrical characterization of heterojunctions was done in the absence of magnetic field (AMF) as well as in presence of magnetic field (PMF).

### Supplementary Information

Supplementary Information.

## Data Availability

All data (XRD, RAMAN,ULTRAFAST,I-V) will be available on nomad-lab on the following id : lUMeM0vTQgSdtAhDTpWm6w. Data will be also available on request to Dr. Pramod Kumar (pkumar@iiita.ac.in).
