# Supplementary material for: Proximity induced band gap opening in topological-magnetic heterostructure (Ni80Fe20/p-TlBiSe2/p-Si) under ambient condition
Source: Sci Rep. 2023 Dec 15;13:22290. doi: 10.1038/s41598-023-49004-5 (PMC10721863; doi:10.1038/s41598-023-49004-5)
Supplement: Supplementary file 1 — Supplementary Information. [file 41598_2023_49004_MOESM1_ESM.pdf]

Supplementary information

Proximity induced band gap opening in topological magnetic heterostructure ($Ni_{80}Fe_{20}$/p-$TlBiSe_2$/p-Si) under ambient condition

Roshani Singh[1], Gyanendra Kumar Maurya[1], Vidushi Gautam[1], Rachana Kumar[2,3], Mahesh Kumar[3],

K. G. Suresh[4], Brahmaranjan Panigrahi[5], Chandrasekhar Murapaka[6], Arbinda Haldar[5], Pramod Kumar[1*]

[1]Spintronics and Magnetic Materials Laboratory, Department of Applied Sciences, Indian Institute of Information Technology Allahabad, Prayagraj 211015 India.
[2]CSIR - Indian Institute of Toxicology Research, Lucknow- 226001 India
[3]CSIR-National Physical Laboratory, New Delhi , India
[4]Department of Physics, Indian Institute of Technology Bombay-400076, India
[5]Department of Physics, Indian Institute of Technology Hyderabad, Kandi 502284, Telangana, India
[6]Department of Materials Science and Metallurgical Engineering, Indian Institute of Technology Hyderabad, Kandi 502284, Telangana, India

*Email ID: pkumar@iiita.ac.in

XRD analysis:

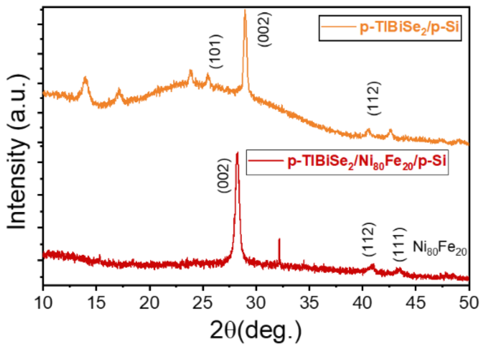

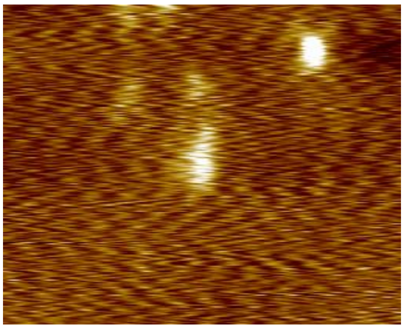

Figure S1: XRD analysis of p-$TlBiSe_2$ film and p-$TlBiSe_2$/$Ni_{80}Fe_{20}$ film deposited on Si substrate .C) AFM scan of p-$TlBiSe_2$/$Ni_{80}Fe_{20}$ /Si surface.

From XRD data, the crystallite size (D) of a $TlBiSe_2$/p-Si film and $TlBiSe_2$/$Ni_{80}Fe_{20}$/p-Si film can be calculated employing Debye Scherrer's equation.

$$D = \frac{k_L \lambda}{\beta \cos\theta} \qquad 1$$

Where k_L represents shape factor (standard value ≈ 0.96), λ is the wavelength of X-ray (1.541A), β is the peak's FWHM in radians and θ is Braggs angle in radians,

Here the crystallite size (D) and strain$(\varepsilon)$of both films were also determined using W-H plot method. The motivation for this method is that the expected solution, for crystallite size (D) and strain extension (E) depends considerably on the Bragg angle defined as[2].

$$\beta_D = \frac{k_L \lambda}{v_D \, Cos\theta} \quad \text{and} \quad \beta_E = C\epsilon \tan\theta \qquad 2$$

If β_D and β_E occur simultaneously, the response is determined by the sum of both components. According to Williamson and Hall's simplification, the cumulative influence can be represented by the entire sum of the squares of these two factors or by a trivial sum.

$$\beta_{total} = \beta_E + \beta_D = C\varepsilon \tan\theta + \frac{k_L \lambda}{v_D \, Cos\theta}$$

Or

$$\beta_{total} \cdot Cos\theta = C\varepsilon \, Sin\theta + \frac{k_L \lambda}{v_D} \qquad 3$$

Where β the full width half maxima (FWHM), K is is Scherrer constant, λ is the wavelength of x rays (1.5406 A^0) andθ is the diffraction angle.

The above equation is a straight-line equation. The relation between sinθ and cosθ gives a straight line with intercept $\frac{k_L \lambda}{v_D}$ and slope C. The line's intercept and slope are employed to calculate crystallite size and strain. The calculated values of crystallite size and lattice strain for p-$TlBiSe_2$/p-Si film are 48.20 nm and 1.33 respectively while in case of p-$TlBiSe_2$/$Ni_{80}Fe_{20}$/p-Si film crystallite size is 9.4 nm and lattice strain is 0. 0186.

Table 1: List of calculated parameters for p-$TlBiSe_2$/p-Si film and p-$TlBiSe_2$/$Ni_{80}Fe_{20}$/p-Si film

**Thin TI film**	**Crystallite size (D)**	**Micro-strains**(σ_n)
p-$TlBiSe_2$/p-Si	48 nm	1.33
p-$TlBiSe_2$/$Ni_{80}Fe_{20}$/p-Si	9.4 nm	0.0186

AFM is the best method for analyzing topological materials because it has an excellent resolution and causes less sample damage while being analyzed therefore in our case the surface morphology was measured using the Park AFM system (NX10) within an effective 5 × 5 μm^2 area (Figures S1C).The roughness of the examined film was found to be 0.124 nm.

Experimental section:

Ultrafast study:

For magnetic field induced ultrafast characterization purpose, another film was deposited to fabricate p-$TlBiSe_2$/$Ni_{80}Fe_{20}$/p-Si heterojunction using the same techniques discussed in the

manuscript In which, on Si substrate firstly $Ni_{80}Fe_{20}$ film is deposited then $TlBiSe_2$ was deposited in top surface of $Ni_{80}Fe_{20}$ (Figure S2). In PMF, ultrafast study shows the ground state splitting resulting to band gap opening in TI material.

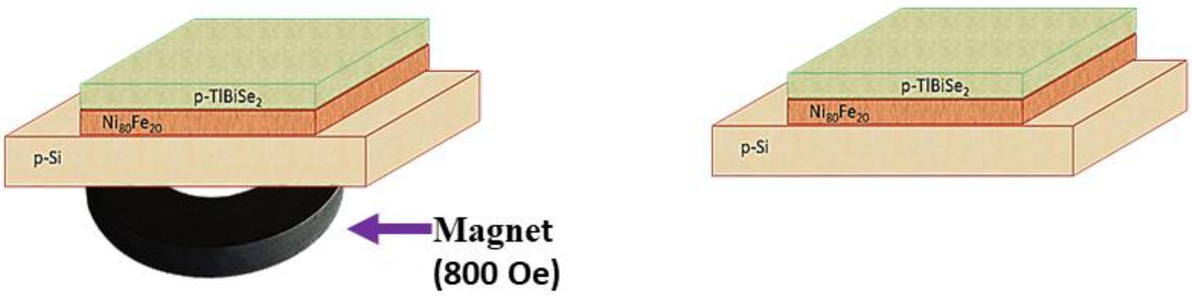

Figure S2: p-$TlBiSe_2$/$Ni_{80}Fe_{20}$/p-Si film in PMF and AMF respectively.

Figure S3 illustrates the schematics of optical pump-probe spectroscopy, in which an array of light pulses emitted by a Ti: Sapphire laser amplifier (35 fs, 4 mJ/pulse,1 KHz, 800 nm) were separated into two different beams via a beam splitter. The beam with high intensity was utilized as a pump or source beam and wavelength of this beam has been varied using an optical parametric amplifier (TOPAS, Light Conversion) in 190 nm to 2600 nm range. On the other hand, the other lower intensity beam is used as probe beam which pass out via CaF_2 crystal to produce white light continuum (WLC) spanning the whole visible spectrum. To optically delay, the probe beam with regard to the pump beam, a computer-controlled delay stage was employed. Along with this, there was a 7fs intrinsic temporal resolution in the delay stage. Here, a pump beam in 490 and 550 nm wavelength range strikes perpendicular and a gated-CMOS detector was used for track the changes in absorption. In order to prevent any adverse effects on TI surfaces, the pump pulse power was carefully kept low (35 mW) for all measurements. But to perform the ultrafast measurement in PMF, the same process was repeated where magnetic field was employed to the sample in perpendicular direction. Using an ultrafast system knows as HELIOS, the time-resolved investigation was carried out.

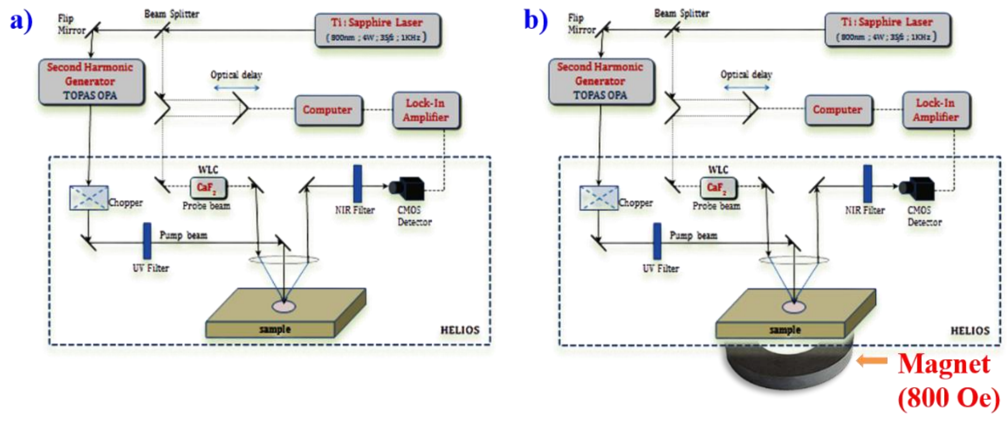

Figure S3: Experimental arrangement for optical Pump probe spectroscopy a) in AMF b) in PMF

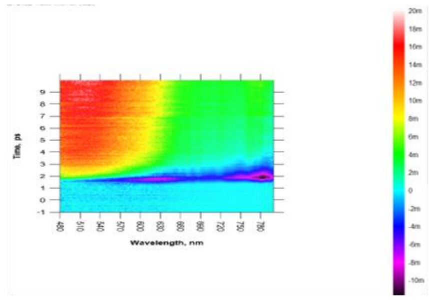

(a) $TlBiSe_2$/p-Si

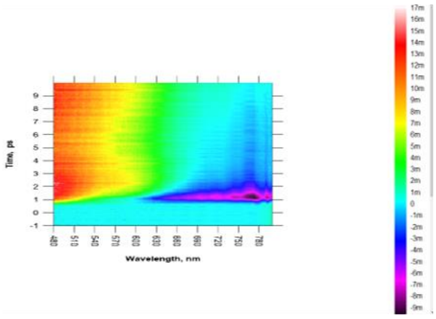

(b) $TlBiSe_2/Ni_{80}Fe_{20}$/p-Si
Without MF

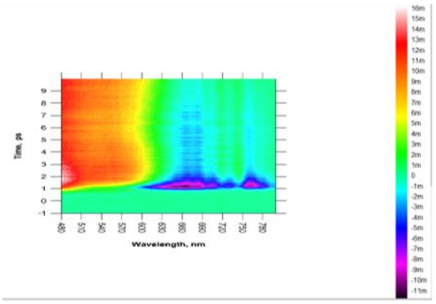

(c) $TlBiSe_2/Ni_{80}Fe_{20}$/p-Si
With MF

Figure S4 represents the ultrafast spectra of (a) $TlBiSe_2$/p-Si (b) $TlBiSe_2/Ni_{80}Fe_{20}$/p-Si in absence of magnetic field and (c) $TlBiSe_2/Ni_{80}Fe_{20}$/p-Si in presence of magnetic field.

From figure S4 it is clear that, in $TlBiSe_2$/p-Si film, there is no splitting in the ground state (figure S4 (a)). While when the $Ni_{80}Fe_{20}$ is incorporated in between the $TlBiSe_2$ and p-Si the Rashba effect comes into the picture. This Rashba effect leads to the small splitting of electronic energy state (figure S4 (b)) having small gap opening. On the other hand when the external magnetic field is applied in $TlBiSe_2/Ni_{80}Fe_{20}$/p-Si film, the ultrafast spectra (figure S4 (c)) revels more splitting of energy states with higher gap opening (around 0.015eV) caused by Zeeman effect.

Again, if we see carefully (By Zooming in) to the Figure S4 (c) the ground state splits into the 3 states and the energy gap between the two successive states is around 0.015 eV (using $E = \frac{hc}{\lambda}$) not exactly 0.015eV because the exact value of lambda in ultrafast surface cannot be located. This energy gap can be verified theoretically as below-

As we know that the Zeeman energy formula is $\Delta E = g\mu_B B \ldots\ldots\ldots\ldots (1)$

Where ΔE represents the energy splitting, g represents the Lande's g factor having value 2 for electrons, μ_B stands the Bohr magneton ($9.274*10^{-24}$ J/T) and B is the applied magnetic field.

From equation (1)

$\Delta E = (2)$*(9.274*10-24 J/T)*(0.08T) because the applied magnetic field is 800 Oe (0.08T)

$$\Delta E \sim 1.86 * 10 - 24\,J$$

Now if want to calculate in unit of eV then

$$\Delta E \sim (1.86 * 10 - 24\,J)/(1.602 * 10 - 19\,\frac{J}{eV})$$

Hence $\Delta E \sim 0.0116\ eV$

.

Electrical analysis under dark:

Under dark condition, the electrical analysis of $Ni_{80}Fe_{20}$/ p-$TlBiSe_2$/p-Si heterojunction was carried out in AMF and PMF both. The obtained result reveled that magnetic field significantly affect the diode parameters.

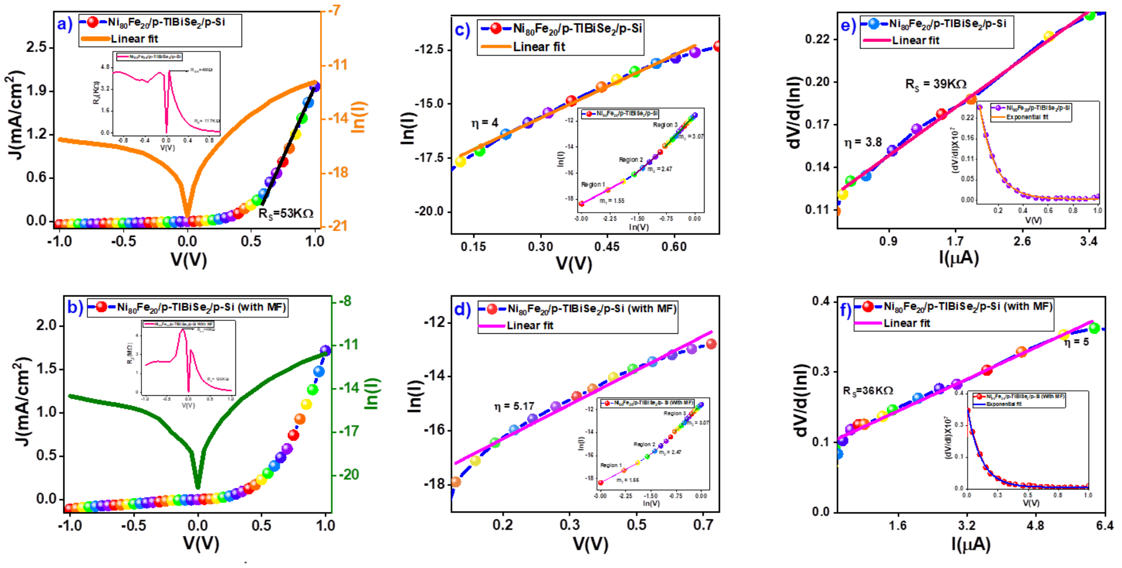

Figure S5: Dark characteristics of $Ni_{80}Fe_{20}$/p-$TlBiSe_2$/p-Si heterostructure at room temperature in AMF and PMF. a) and b) show the current density vs. voltage (J-V) and magnified semi log characteristics plot, while the inset shows resistance voltage (R-V) plot in AMF & PMF respectively. c) and d) shows the semi – log (I) *vs* V characteristics while inset shows the log(I) *vs* log(V) plots indicating different parts of conduction mechanism with different linear fittings, in AMF & PMF respectively. e) and f) show the forward – biased linearly fitted plot of $\frac{dV}{d(lnI)}$ *vs.* I and the inset shows the slope – voltage ($\frac{dV}{dI}$) *vs.* V characteristics, revealing the exponential relation of the slope with applied voltage in AMF and PMF.

Electrical analysis under illumination:

The electrical analysis of examined heterostructure was done under ambient condition using Kiteley 4200**.** Figure S6a & S6b show J-V characteristics of examined heterojunction under light effect in AMF and PMF respectively. The results demonstrate excellent photoelectric and photovoltaic effect in both forward and reverse bias regions. The photodetection efficiency of device was assessed through performance parameters like, photo to dark current ratio $PDCR = I_{ph}/I_{dark}$, photoresponsivity$R = I_{ph}/P_i$, detectivity $D = RA^{1/2}/\sqrt{2qI_{dark}}$, sensitivity $S = R(d/V_d)$ and photoconductive gain $G = Rh\nu/q\eta^3$, where $I_{ph} = (I_{light} - I_{Dark})$ is photo current , I_{dark} is dark current, P_i represent the power density of laser light , A represents the effective area of device for absorbing incoming light (0.0049 cm^2), ν represents the frequency of the incoming laser light, h is the Planck's constant , η is the external quantum efficiency, V_d is the bias voltage and d is the thickness of the diode(~150 nm).

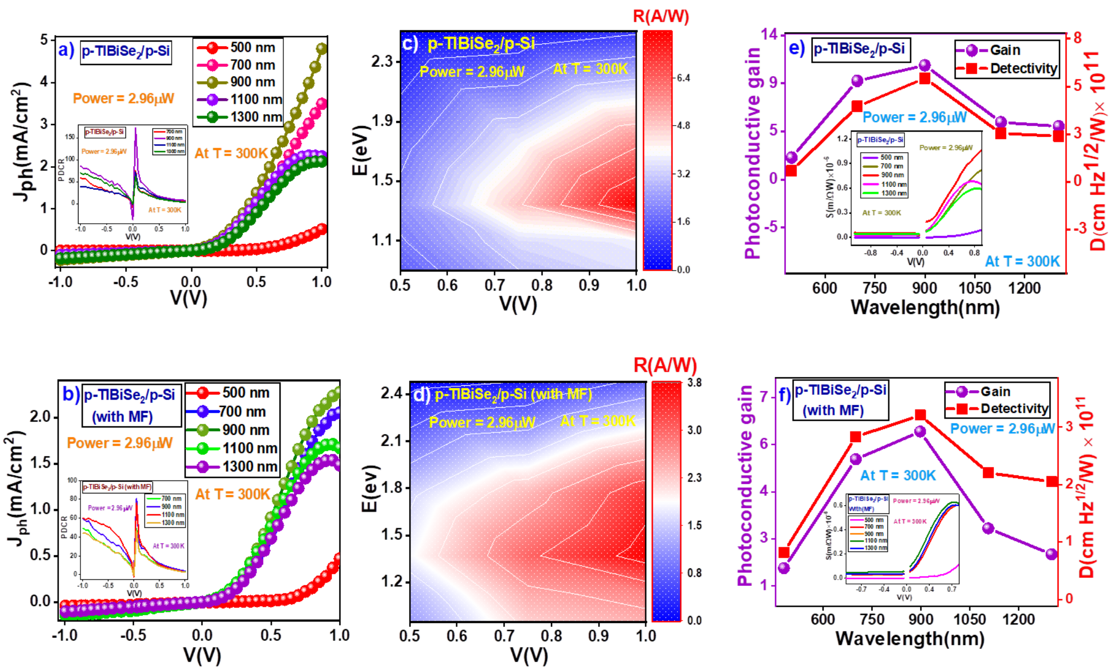

Figure S6: Optoelectronic characteristics of p-$TlBiSe_2$/p-si heterojunction under varying wavelengths from 500 to 1300 nm at 2.96 µW power, in AMF and PMF. a) and b) show the Jph-V characteristics in AMF and PMF field respectively while corresponding insets show the PDCR vs voltage characteristics. c) and d) show the Energy- Responsivity (E-R) vs voltage (V) contour in AMF and PMF respectively. e) and f) show the photoconductive gain (G) -detectivity (D) vs wavelength plot while corresponding insets show the sensitivity vs voltage plot in AMF and PMF respectively

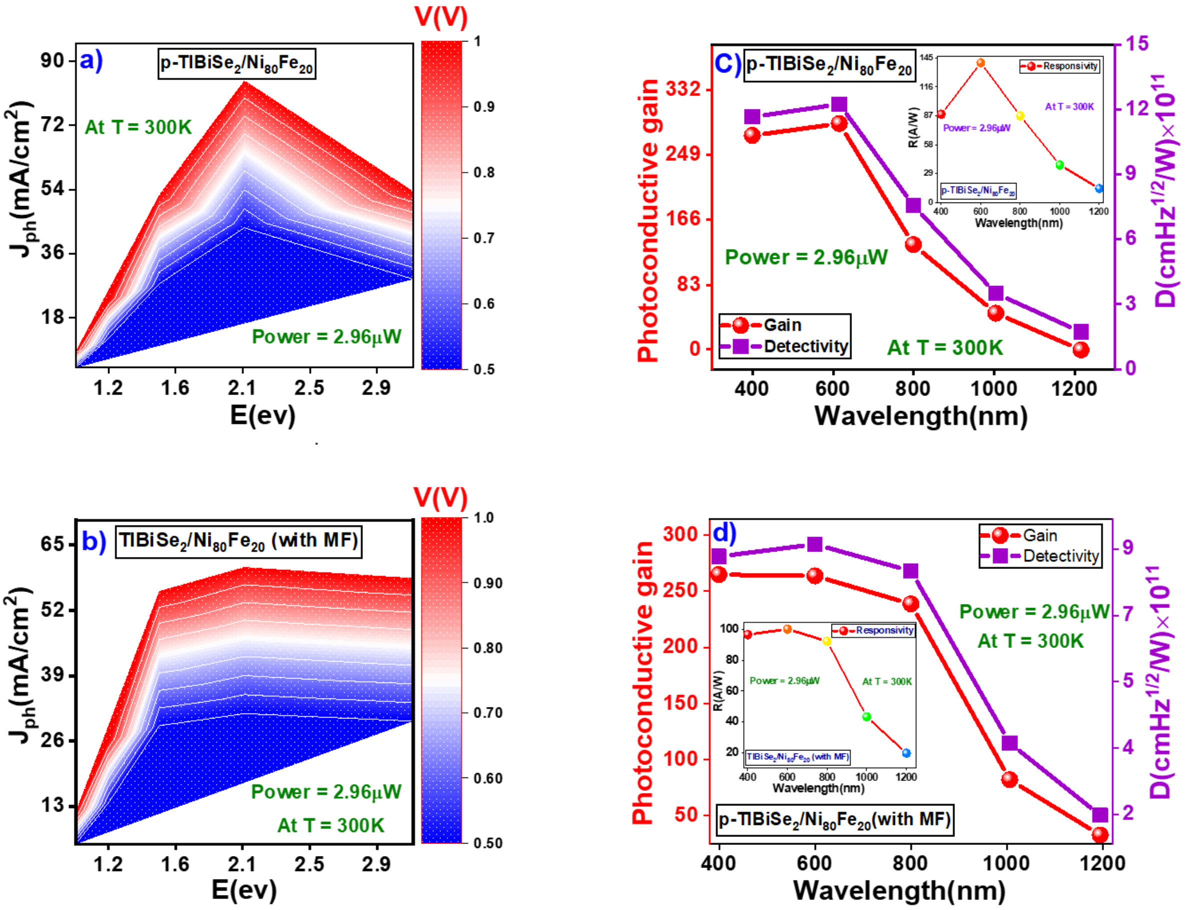

Figure S7; Optoelectronic characteristics of p-$TlBiSe_2/Ni_{80}Fe_{20}$heterojunction under varying wavelength from 400 to 1200 nm at 2.96μW power, in AMF and PMF. a) and b) depict the photocurrent density-Energy (J_{ph}-E) contour plot against varying bias voltage in AMF and PMF respectively. c) and d) depict the photoconductive gain-detectivity (G-D) vs. wavelength plot while corresponding insets show the responsivity vs. wavelength in AMF and PMF respectively.

All the performance parameters for p-$TlBiSe_2/Ni_{80}Fe_{20}$ heterojunction are listed in the following table.

Table 2: List of photo detection parameters at +1V corresponding to 600 nm wavelength for $TlBiSe_2/Ni_{80}Fe_{20}$ heterojunction in AMF and PMF.

Device	Wavelength (nm)	(I_F)μA	R(A/W)	G	D (Jones)×10^{12}
$TlBiSe_2/Ni_{80}Fe_{20}$ (AMF)	600	411	140.18	290	12.25
$TlBiSe_2$/ $Ni_{80}Fe_{20}$ (PMF)	600	122	100.378	280	9.12

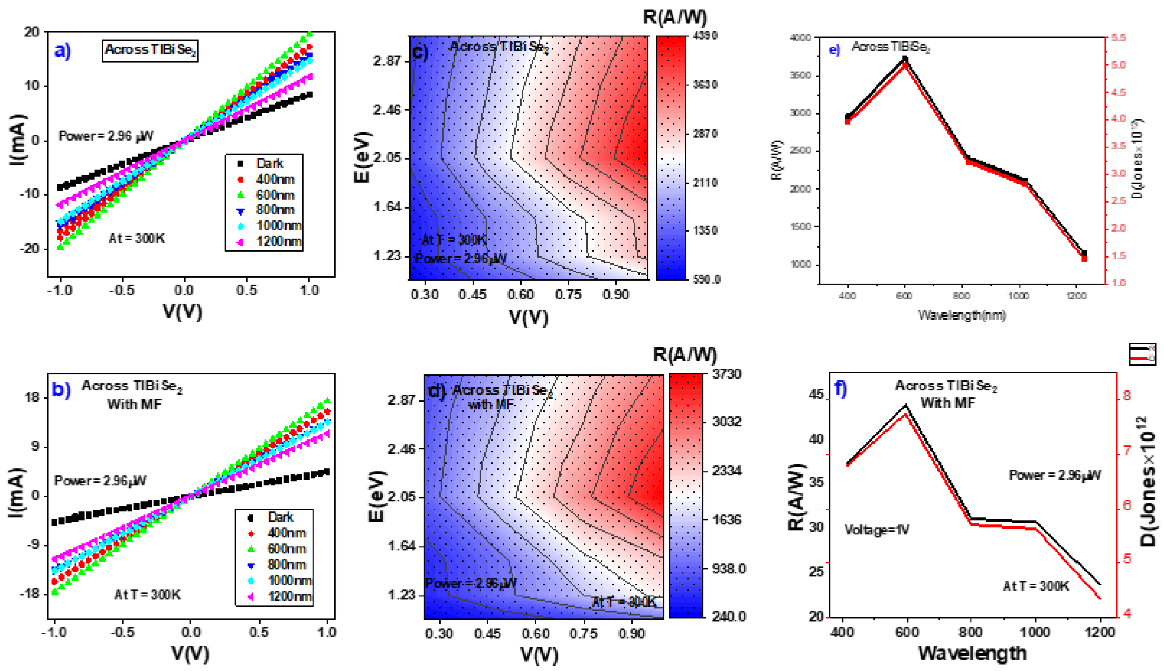

Figure S8: Optoelectronic characteristics across p-$TlBiSe_2$ under varying wavelength from 400 to 1200 nm at 2.96μW power, in AMF and PMF. a) and b) depict the current -voltage(I-V) plot in AMF and PMF respectively. c) and d) show the energy -responsivity vs voltage contour plot in AMF and PMF respectively. e) and f) depict the responsivity-detectivity (R-D) vs wavelength plot in AMF and PMF respectively.

Table 3: List of photo detection parameters at +1V corresponding to 600 nm wavelength across $TlBiSe_2$ in AMF and PMF.

Device	Wavelength (nm)	(I_F) (mA)	R(A/W)	D (Jones)×10^{12}
Across$TlBiSe_2$ (AMF)	600	20	2450	7.9
Across $TlBiSe_2$ (PMF)	600	16.5	1847	5

2 diode model:

The two-diode model is frequently used to illustrate the dark and illuminated current-voltage (I-V) behavior of a p-n junction diode, with the first diode representing the current due to charge carrier diffusion, having ideality factor one, and the second diode reflecting the current due to recombination in the depletion region. The second diode component commonly has an ideality factor of two, though the ideality factor for several p-n junction diodes is greater than two. Figure S9 depicts a schematic of a 2-diode model which was used to simulate the examined heterojunction using two diodes[45]. The current source generates the photocurrent J_{Ph}, indicating illumination. The circuit can be used to display dark J-V measurements by setting J_{Ph}=0. The experimental I-V results of thep-$TlBiSe_2$/p-Si

heterojunction and $Ni_{80}Fe_{20}$/p-$TlBiSe_2$/p-Si heterojunction were utilized to simulate a 2-diode model[67]. J(V) and η(V) in figure S9 indicate the current density and voltage at the contacts, correspondingly. Rpp and Rss are the series and parallel resistances built into junction devices which contain all the generating currents like diffusion, recombination, and ohmic[89]. In the depletion region, diode D_{01} indicates the diffusion current $J_d(V)$, while diode D_{02} indicates the recombination current Jr(V).

$$J(V) = J_{01}\left(e^{\frac{qV}{n_1kT}} - 1\right) + J_{02}\left(e^{\frac{qV}{n_2kT}} - 1\right) = J_d(V) + J_r(V)$$

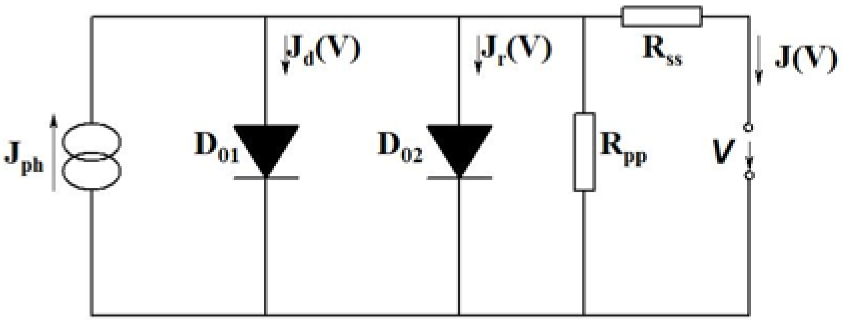

Figure S9: 2 diode model circuit used to simulate TlBiSe2/p-Si heterojunction diode and $Ni_{80}Fe_{20}$/p-$TlBiSe_2$/p-Si heterojunction diode in AMF and PMF under dark and illumination conditions.

J_{01} and J_{02} are the pre-exponential components of $J_d(V)$ and Jr(V) respectively. J_{01} is the recombination current that exists in the bulk and typically becomes dominant at higher bias. J_{02} reflects the saturation current density caused by depletion region recombination at the junction, and it becomes dominant at smaller bias. n_1, n_2 are the ideality factors for diodes D_{01} and D_{02}.The ideal value of n_1 and n_2 is 1 and 2 respectively[1011]. The 2-diode model was used to fit and determine numerous parameters, including J_{01}, J_{02}, n_1, n_2, R_{pp} and Rss. The fitting results achieved following the 2-diode simulation of the experimental results observed for the p-TlBiSe2/p-Si heterojunction diode and $Ni_{80}Fe_{20}$/p-$TlBiSe_2$/p-Si heterojunction diode are shown in Figure S10. Figure S10 illustrates the well fitted acquired current density-voltage (J-V) plot.

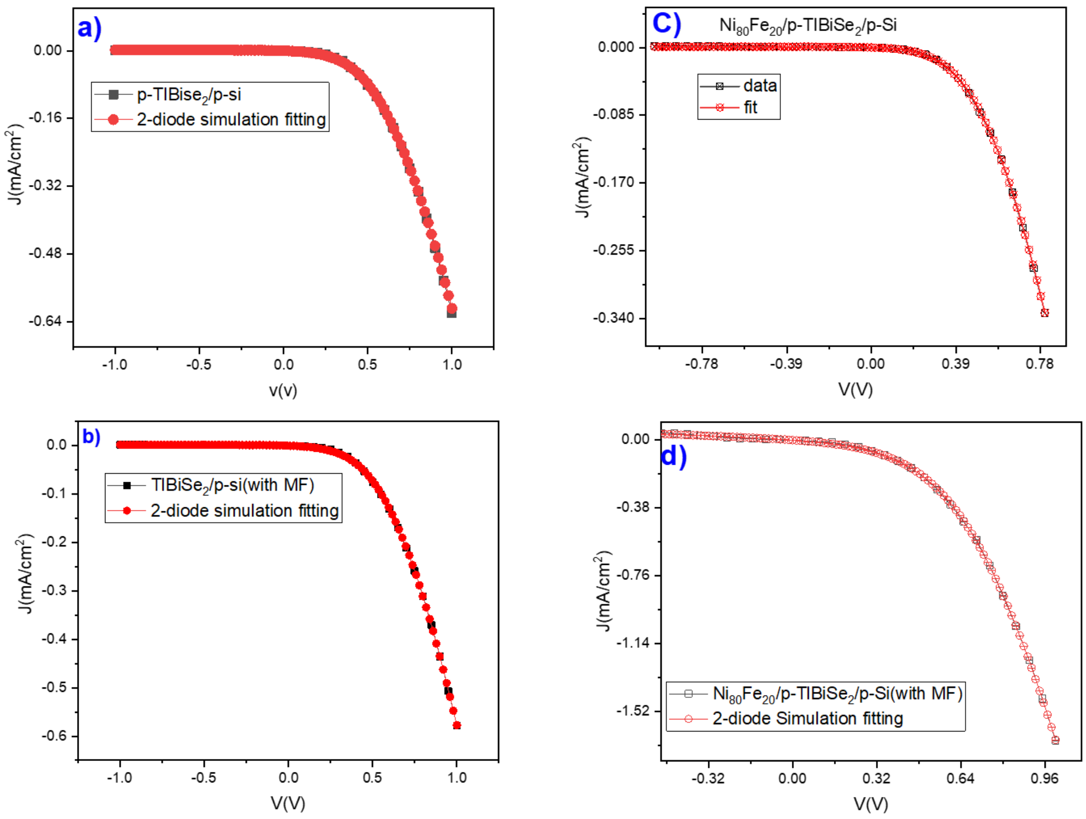

Figure S10: Current density vs. voltage (J-V) plot simulated by 2 diode model in dark. a) and b) for TlBiSe2/p-Si heterojunction diode in AMF and PMF respectively. c) and d) for $Ni_{80}Fe_{20}$/p-$TlBiSe_2$/p-Si heterojunction diode in AMF and PMF respectively.

Hall effect measurement:

The Hall measurement of $TlBiSe_2$ and $Ni_{80}Fe_{20}$ film was carried out under ambient condition to determine the charge transport parameters using four probe Van der Pauw method (8400 series, Lake Shore CRYOTRONICS). Before the Hall measurement, the suitable contacts in both films was deposited via Thermal evaporator. Now to perform the Hall measurement, the sample is loaded in Sample card having five 10 mm solder pad sample card (four without sensor and one with platinum sensor). This sample card is mounted in “Standard insert” which provides electrical connections to the sample card. The standard insert is carefully inserted in “Light Tight body” which provides a draft free environment to the sample also it is designed to fit in magnetic air gap and the sample is ready for the measurement. Now go to the Lake Shore software, select the type of your sample such as van der Pauw. Then provide some details like thickness and other dimensions and supply some current to the sample. After that give current to the electromagnets also using the power supply. This current in the electromagnets generates a magnetic field in the perpendicular direction. Due to this

magnetic field, the potential difference between two opposite sides of sample generates, which is known as Hall voltage. By the help of this developed hall voltage many charge transport parameters like carrier concentration, type of the charge carries can be calculated very easily. In our work the obtained Hall measurement results of p-$TlBiSe_2$ film confirms p type carriers having a concentration of 6.17 x $10^{26}/m^3$ and Hall mobility 3.7 x $10^{-3} cm^2/V$-s while the measurement on $Ni_{80}Fe_{20}$ heterostructure shows a carrier concentration of $9.12\times10^{32}/m^3$.

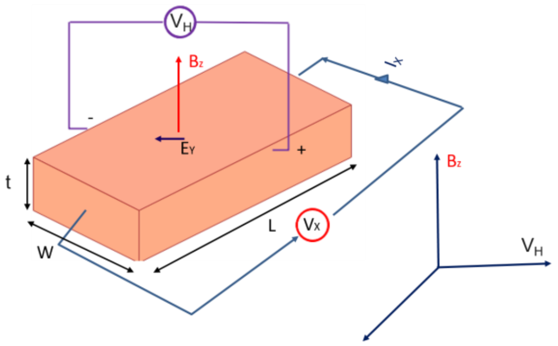

(a)

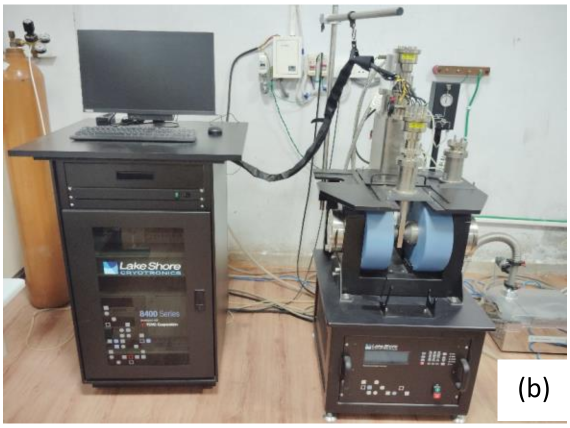

(b)

Figure S11(a) shows the pictorial representation of Hall effect and figure S11(b) represents the Lake Shore Hall measurement system.

Raman and Ultrafast study on $Ni_{80}Fe_{20}$/p-Si:

The figure S12 represents the Raman spectra of $Ni_{80}Fe_{20}$/p-Si and p-$TlBiSe_2$/$Ni_{80}Fe_{20}$/p-Si heterostructure. Figure S13 shows the ultrafast study of $Ni_{80}Fe_{20}$/p-Si heterostructure in Visible and NIR region. The obtained results indicate that there is no change in signal in Raman and ultrafast study of $Ni_{80}Fe_{20}$/p-Si heterostructure.

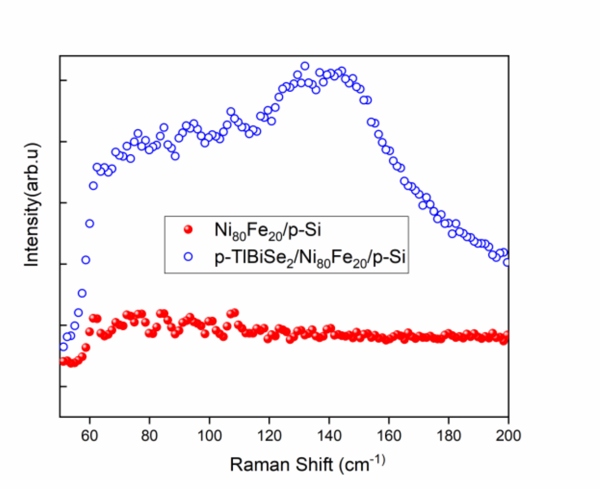

Figure S12: Raman spectra of (a) $Ni_{80}Fe_{20}$/p-Si (b) p-$TlBiSe_2$/$Ni_{80}Fe_{20}$/p-Si heterostructure.

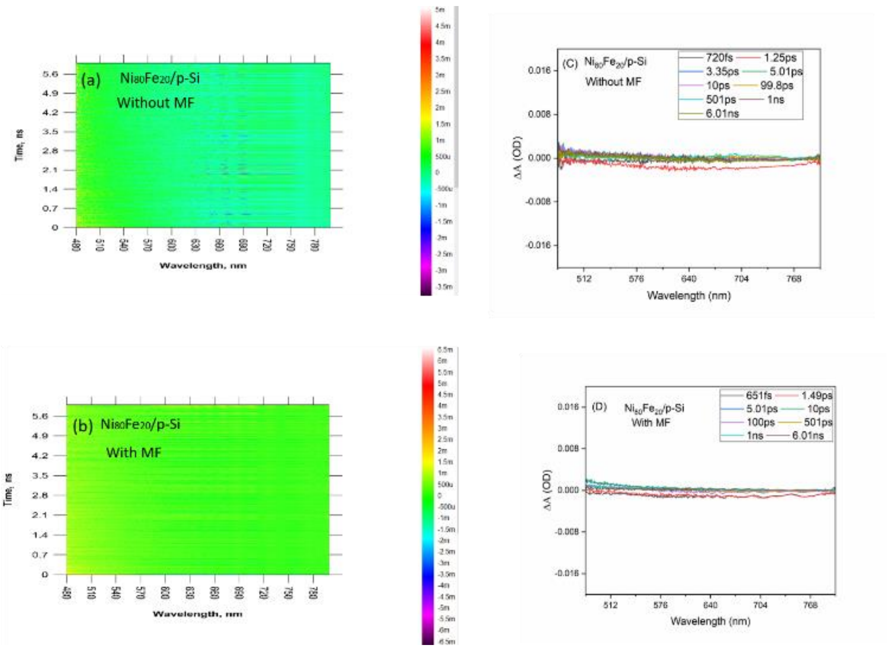

Figure S13(a) and S13(b) represents the ultrafast surface of $Ni_{80}Fe_{20}$/p-Si in absence and presence of magnetic field respectively.

Magnetotrasport study:

The magnetoresistence behavior of $TlBiSe_2/Ni_{80}Fe_{20}$/p-Si film is shown in figure S14 (a) while the figure S14 (b) shows the magnetoresistence of $TlBiSe_2$ on Si. Here both MR curve shows the cusp nature while in case of $TlBiSe_2$/p-Si, the cusp is sharper than $TlBiSe_2/Ni_{80}Fe_{20}$/p-Si film. The MR curve of $TlBiSe_2$/p-Si film has a nature something close to linear while in $TlBiSe_2/Ni_{80}Fe_{20}$/p-Si film, it follows a kind of parabolic nature. This can be a result of breaking of time reversal symmetry and destruction of linear dispersion relation in surface states of topological material. As the magnetic field breaks the symmetry of electron motion, the resistance varies parabolically.

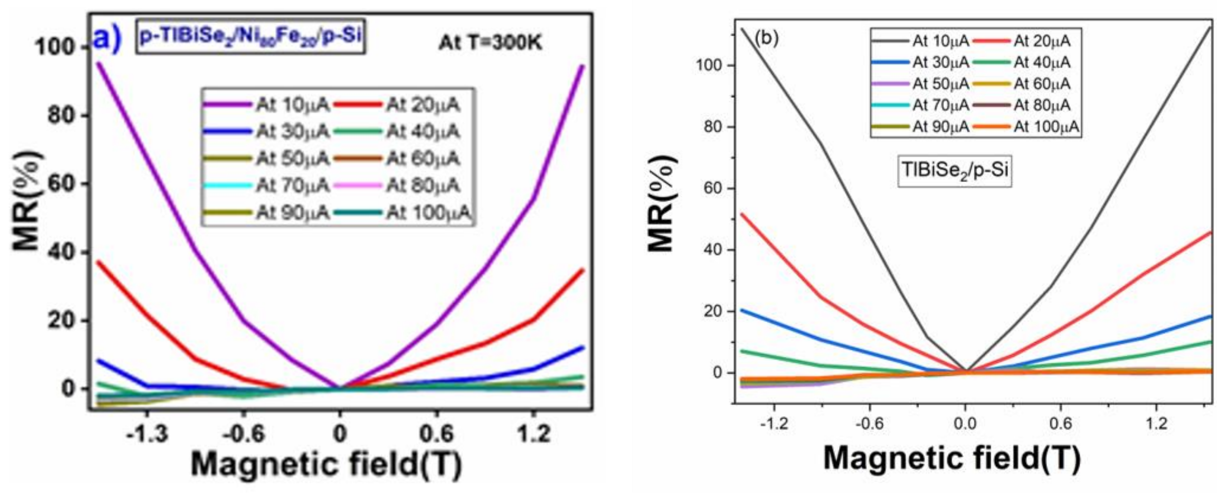

Figure S14: shows the Magnetoresistence curve of (a) $TlBiSe_2/Ni_{80}Fe_{20}$/p-Si film and (b) $TlBiSe_2$/p-Si film

The magnetoresistance behavior of a $TlBiSe_2/Ni_{80}Fe_{20}$/p-Si film is influenced by the properties of both materials ($TlBiSe_2$ & $Ni_{80}Fe_{20}$) and their interfaces. When $TlBiSe_2$ is deposited over $Ni_{80}Fe_{20}$, the ferromagnetic properties of $Ni_{80}Fe_{20}$ and the unique electronic states of $TlBiSe_2$ can interact. The magnetization dynamics in $Ni_{80}Fe_{20}$ have the potential to influence the magnetic field experienced by the $TlBiSe_2$ layer, thereby influencing the cyclotron motion of charge carriers within the topological surface states. The observed magnetoresistance behavior in $TlBiSe_2/Ni_{80}Fe_{20}$/p-Si heterostructure can be attributed to the combined effect of quantized cyclotron orbits in $TlBiSe_2$ and the magnetic properties of $Ni_{80}Fe_{20}$. The variations in the magnetic field can potentially induce changes in the cyclotron motion of charge carriers within $TlBiSe_2$, thereby having an influence on the overall

resistance of the heterojunction. As the role of the topological surface states of $TlBiSe_2$ is of significant importance in the magnetoresistance observed in the $TlBiSe_2/Ni_{80}Fe_{20}$/p-Si film and these states exhibit quantized cyclotron orbits. In short, the magnetoresistance observed in $TlBiSe_2/Ni_{80}Fe_{20}$/p-Si film can be modulated by the cyclotron motion exhibited by the charge carriers in the topological surface states of $TlBiSe_2$ Hence MR can be a form of "cyclotronic magnetoresistance."

The cusp in the $TlBiSe_2$ /Ni $_{80}$ Fe_{20} /p-Si film is (corresponding to 10μA current) = 0.20*= 0.00775(approx.)

The cusp in the $TlBiSe_2$ /p-Si film is (corresponding to 10μA current) = 0.30*= 0.0116(approx.)

Bibliography:

1. Devonport, A. *et al.* Magnetic properties of chromium-doped Ni80Fe20 thin films. *J. Magn. Magn. Mater.* **460**, 193–202 (2018).

2. Jabir, S. A.-A. & Harbbi, K. H. A comparative study of Williamson-Hall method and size-strain method through X-ray diffraction pattern of cadmium oxide nanoparticle. in 020015 (2020). doi:10.1063/5.0033762.

3. Caglar, M. Electrical and photovoltaic properties of heterojunction diode based on poly(3,4-ethylenedioxythiophene):poly(styrenesulfonate). *Eur. Phys. J. Appl. Phys.* **60**, 30102 (2012).

4. Gupta, S., Tiwari, H., Fozdar, M. & Chandna, V. Development of a Two Diode Model for Photovoltaic Modules Suitable for Use in Simulation Studies. in *2012 Asia-Pacific Power and Energy Engineering Conference* 1–4 (IEEE, 2012). doi:10.1109/APPEEC.2012.6307201.

5. Weidong Xiao, Dunford, W. G. & Capel, A. A novel modeling method for photovoltaic cells. in *2004 IEEE 35th Annual Power Electronics Specialists Conference (IEEE Cat. No.04CH37551)* 1950–1956 (IEEE). doi:10.1109/PESC.2004.1355416.

6. Suckow, S., Pletzer, T. M. & Kurz, H. Fast and reliable calculation of the two-diode model without simplifications. *Prog. Photovoltaics Res. Appl.* **22**, 494–501 (2014).

7. Breitenstein, O. & Rißland, S. A two-diode model regarding the distributed series resistance. *Sol. Energy Mater. Sol. Cells* **110**, 77–86 (2013).

8. Sawada, M. *et al.* Electrical characterization of n-GaN Schottky and PCVD-SiO2/n-GaN interfaces. *J. Cryst. Growth* **189**–**190**, 706–710 (1998).

9. Breitenstein, O. Nondestructive local analysis of current–voltage characteristics of solar cells by lock-in thermography. *Sol. Energy Mater. Sol. Cells* **95**, 2933–2936

(2011).

10. Rivas, N. *et al.* Generation and detection of coherent longitudinal acoustic waves in ultrathin 1 <i>T'</i> -MoTe $_2$. *Appl. Phys. Lett.* **115**, 223103 (2019).

11. Segawa, K. Synthesis and characterization of 3D topological insulators: a case TlBi(S $_{1-x}$ Se $_x$) $_2$. *Sci. Technol. Adv. Mater.* **16**, 014405 (2015).
